# CombiANT: Antibiotic interaction testing made easy

**DOI:** 10.1371/journal.pbio.3000856

**Published:** 2020-09-17

**Authors:** Nikos Fatsis-Kavalopoulos, Roderich Roemhild, Po-Cheng Tang, Johan Kreuger, Dan I. Andersson

**Affiliations:** 1 Department of Medical Biochemistry and Microbiology, Uppsala University, Uppsala, Sweden; 2 Department of Medical Cell Biology, Uppsala University, Uppsala, Sweden; Universitat zu Koln, GERMANY

## Abstract

Antibiotic combination therapies are important for the efficient treatment of many types of infections, including those caused by antibiotic-resistant pathogens. Combination treatment strategies are typically used under the assumption that synergies are conserved across species and strains, even though recent results show that the combined treatment effect is determined by specific drug–strain interactions that can vary extensively and unpredictably, both between and within bacterial species. To address this problem, we present a new method in which antibiotic synergy is rapidly quantified on a case-by-case basis, allowing for improved combination therapy. The novel CombiANT methodology consists of a 3D-printed agar plate insert that produces defined diffusion landscapes of 3 antibiotics, permitting synergy quantification between all 3 antibiotic pairs with a single test. Automated image analysis yields fractional inhibitory concentration indices (FICis) with high accuracy and precision. A technical validation with 3 major pathogens, *Escherichia coli*, *Pseudomonas aeruginosa*, and *Staphylococcus aureus*, showed equivalent performance to checkerboard methodology, with the advantage of strongly reduced assay complexity and costs for CombiANT. A synergy screening of 10 antibiotic combinations for 12 *E*. *coli* urinary tract infection (UTI) clinical isolates illustrates the need for refined combination treatment strategies. For example, combinations of trimethoprim (TMP) + nitrofurantoin (NIT) and TMP + mecillinam (MEC) showed synergy, but only for certain individual isolates, whereas MEC + NIT combinations showed antagonistic interactions across all tested strains. These data suggest that the CombiANT methodology could allow personalized clinical synergy testing and large-scale screening. We anticipate that CombiANT will greatly facilitate clinical and basic research of antibiotic synergy.

## Abstract 2

Existing methods for identifying efficient combinations of antibiotics are time-consuming and costly, restricting their use in clinics and research. This study describes the novel CombiANT methodology, which uses defined diffusion landscapes of three antibiotics to permit rapid and low-cost synergy quantification between all antibiotic pairs.

## Introduction

Antibiotic therapy increasingly relies on the combined activity of 2 or more agents. Combinations of antibiotics are applied for up to 50% of patient cases in the treatment of severe surgical site infections, bacteremia, pneumonia, or septic shock [1–4]. Antibiotic combinations are also frequently prescribed as prophylactic treatments to prevent post-operation-related infections [5,6] and in the treatment of complicated chronic infections [7–9]. The rationale for combination therapy compared to monotherapy is 3-fold: (i) a broadened activity range by combining the different modes of action, pharmacodynamics, and pharmacokinetics of different antibiotics, (ii) stronger treatment effect, and (iii) reduced risk of resistance evolution. The spread of antibiotic resistance has put an emphasis on the latter property, in that treatments need to be effective despite preexisting resistance and emergence of resistance during treatment needs to be prevented [10–12]. Combinations of certain antibiotics show high efficacy against resistant pathogens [13], and several cases exist in which combinations allow treatment of, e.g., vancomycin-resistant *Staphylococcus aureus* (VRSA) [14], resistant bacteria that express beta-lactamase [15], and heteroresistant pathogen populations that are refractory to killing by a single antibiotic [16]. Moreover, antibiotic combinations often reduce the rate of resistance evolution during treatment, especially for chronic infections, as highlighted by *P*. *aeruginosa* in cystic fibrosis and other bacterial pathogens [10,17–19]. The efficacy of these multitarget treatments is likely explained by a requirement for the co-occurrence of multiple resistance factors for treatment escape.

The individual inhibitory effects of antibiotics on the bacterial cell can interact to produce combination effects that are either stronger (synergistic) [20,21] or weaker (antagonistic) [22] than expected from additivity. Antagonistic and especially suppressive drug interactions could reduce the treatment effect, and, conversely, positive synergistic activities could provide an edge in refined combination therapy. Thus, the proper use of combination therapy hinges upon our ability to quantify the synergy profile of antibiotic combinations.

Synergy and antagonism are not inherent properties of an antibiotic pair but arise from the combination of actions that the antibiotics have on bacterial cells. Thus, the interaction, be it positive, additive, or negative, is the net effect of drug-induced damage and drug-defense responses. As a result, variation in the genetics and physiology of the bacterial population could potentially cause variation in antibiotic interaction type. Indeed, several recent studies have shown that co-treatment with different pairs of clinically relevant antibiotics exhibit interaction patterns that depend both on the bacterial species and the specific strain within a species [23–26]. Together, these data strongly support the idea that antibiotic interactions depend on genetic variation in the bacterium, which is currently not considered for combination therapy in clinical settings.

Refined combination therapy requires a case-by-case examination of antibiotic interactions for each patient isolate. However, such personalized diagnostics is—because of its complexity and high labor cost—not feasible with the current gold-standard methods, broth-based checkerboard assays and time-kill experiments [27,28]. Agar plate-based methods are easier to perform and multiplex but suffer from other drawbacks. Antibiotic disk diffusion can be used for the qualitative detection of strong interactions between antibiotics, provided that multiple disks loaded with different antibiotics are placed in sufficient proximity to generate areas of an agar plate with multiple antibiotics acting together [29,30]. The shape of the combined inhibition zone provides a qualitative indication of synergy or antagonism. An extension of the disk diffusion generates more defined areas of antibiotic combination gradients using cross-formation of antibiotic gradient strips (Epsilometer tests) [27] or filter paper strips [31]. There are, however, some drawbacks of using these assays. Gradient strips need to cross each other at their minimal inhibitory concentration (MIC) values for the assay to allow a calculation of the combinatory effect, while the filter paper strips need to be removed after application and cannot be part of the plate while inoculating. Although these methods produce quantitative measurements, they are restricted to 2 antibiotics per plate and require advance knowledge of the MICs of the antibiotics tested. An assay that could produce quantitative measurements of antibiotic synergy while still remaining simple enough to use would ideally serve the needs both of clinical and academic laboratories alike.

In this study, we present a new and easy-to-conduct assay for testing antibiotic synergy that is robust and highly quantitative. Our solution, CombiANT, is a diffusion-based assay that provides quantitative information of all pairwise interactions of 3 antibiotics in a single agar plate. The present study shows that CombiANT performs equally well to broth-based checkerboard methodology, but due to its unique design and function, offers much reduced method complexity that is comparable to a disk diffusion test. Similar to checkerboard assays, CombiANT produces fractional inhibitory concentration indices (FICis) but enables higher throughput. The assay can be applied without previous information of the susceptibility of the strain. We demonstrate the potential of CombiANT for antibiotic interaction screening by applying the assay to the antibiotics used for treating *E*. *coli* urinary tract infections (UTIs). Lastly, we identified conserved and variable antibiotic interactions that have high potential for personalized and refined combination therapy.

## Results

### CombiANT assay and system design

**Fig 1:**
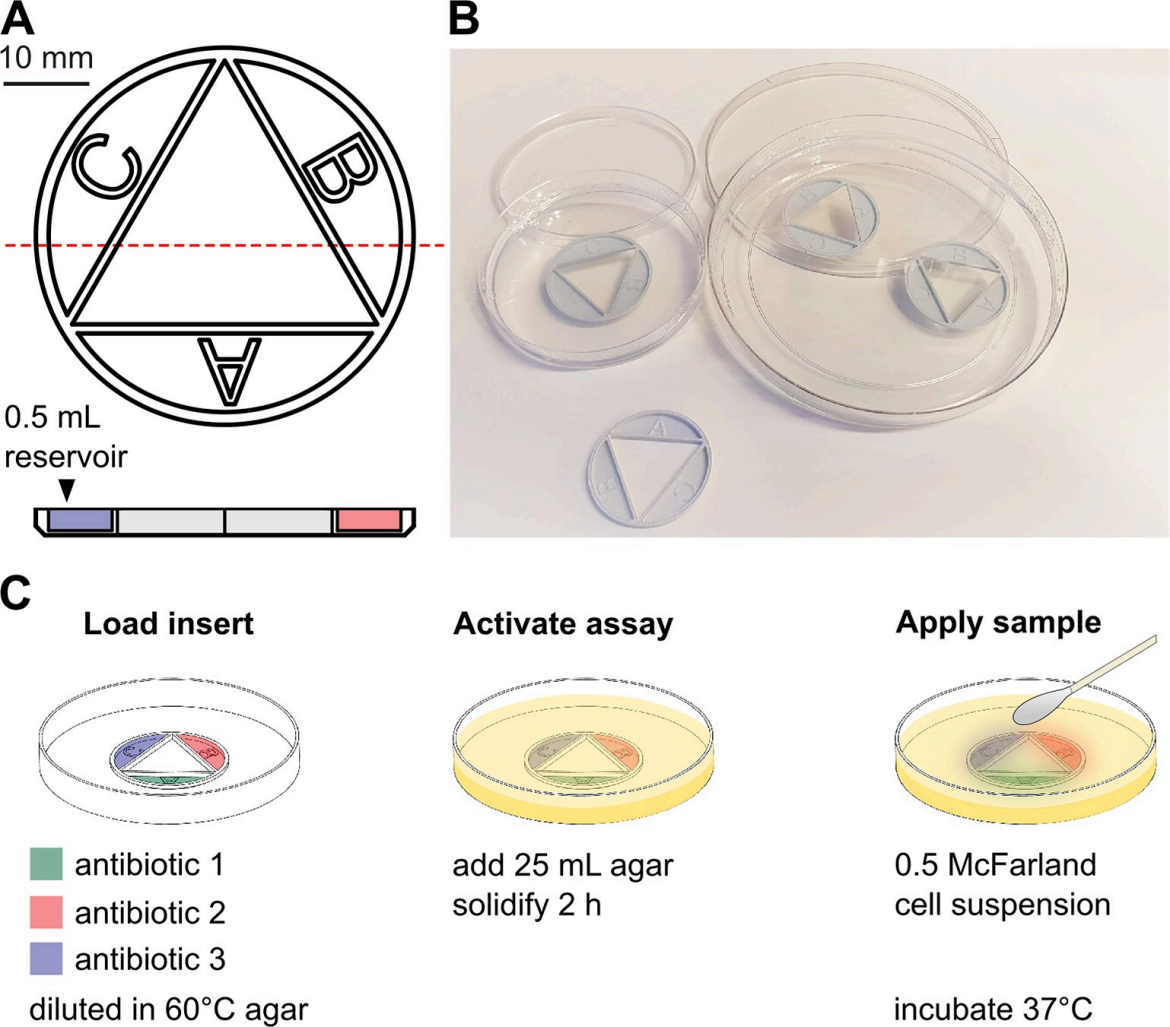
Design of the assay and the experimental protocol. (A) Technical drawing of the CombiANT insert with top view (top) and side projection along the red dashed line (bottom). The insert comprises 3 antibiotic reservoirs (marked ‘A,’ ‘B,’ and ‘C’) that surround an internal triangular area called the interaction imaging area. The outer diameter of the insert is 42 mm. (B) Examples of how CombiANT assays can be used, whether in multiples or single assays in standard agar plates. (C) Illustration of the assay protocol. The insert is loaded by adding 0.5 mL liquid agar (60°C) containing antibiotics into the reservoirs. The prepared inserts can be stored at 4°C–8°C. To activate an insert, a second layer of 25 mL agar is added as to enclose the insert and fill the plate, thereby permitting diffusion of the antibiotics to the agar surface and the reservoir periphery. After solidification, a bacterial cell suspension of 0.5 McFarland is inoculated on the agar surface using a sterile cotton swab and exposed to the antibiotic gradient landscape. The finished plates are incubated at 37°C, and stable zones of growth inhibition establish within 16–24 hours. Numerical values for this figure are available in S1 Data.

The CombiANT assay was designed to fulfil the following criteria: (i) the generation of quantitative information of antibiotic interactions, (ii) a reduction of assay complexity and work hours for assay preparation and analysis, (iii) high throughput, and (iv) easy integration into clinical microbiology laboratory routines. Our solution is a diffusion-based assay that provides quantitative information of the pairwise synergy of 3 different antibiotics in a single agar plate. The assay consists of a custom-designed culture inserts (Fig 1A) that can be integrated into any standard cell culture plate (Fig 1B). Multiple inserts can be used on the same plate (Fig 1B). The insert comprises 3 reservoirs (marked ‘A,’ ‘B,’ and ‘C’) housing antibiotics and a central triangular interaction area (Fig 1A).

To run a CombiANT assay, antibiotic-containing agar is loaded into the insert reservoirs by pipetting (Fig 1C). For most applications in this study, the reservoirs are loaded with different antibiotics. Upon agar solidification, the assay is in an inactive state. At this point, the inserts can be stored under refrigeration, with no loss of function for at least 1 week (depending on the antibiotics used). This allows for multiple assays, encompassing different antibiotics, to be prepared and stored according to the user’s need so that they can be easily used without delay.

**Fig 2:**
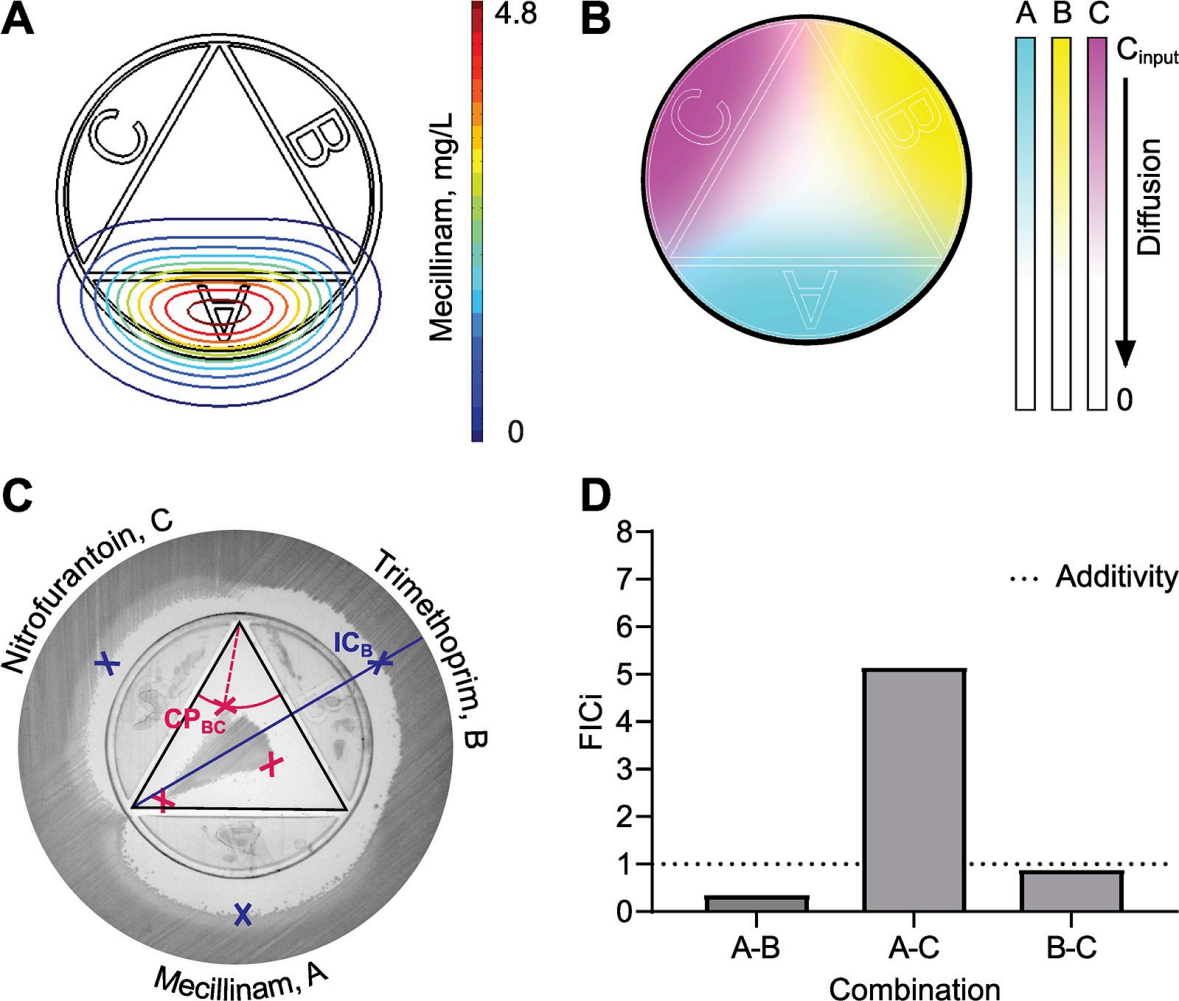
Assay technology and synergy readout. (A) Diffusion map for the antibiotic MEC after 24 hours. Lines indicate equal surface concentrations. The chromatic scale represents concentration of MEC starting the input concentration of 4.8 mg/L denoted in red, down to negligible concentrations in blue. (B) Agar surface antibiotic diffusion landscape for 3 antibiotics. The chromatic scale represents concentrations of antibiotic starting from input concentrations with blue indicating antibiotic A, yellow indicating antibiotic B, and pink indicating antibiotic C, down to negligibly low concentrations expressed by white. (C) Description of CombiANT analysis using a representative assay result. The *E*. *coli* strain DA46056 that was isolated from a UTI was here tested against the antibiotics MEC, TMP, and NIT. Inhibition zones have formed around the antibiotic reservoirs because of the antibiotic diffusion. The black triangle indicates the interaction imaging area. Blue points outside of the interaction imaging area indicate the IC points in the agar surface of the antibiotics acting alone. As illustrated by the blue line and IC_B_, the blue IC points are placed at the midpoint of the outer inhibition zone from the corresponding reservoir, here reservoir B. Red points inside the interaction imaging area indicate CPs of adjacent antibiotic pairs. As illustrated by the red lines and CP_BC_, the red CP points are placed at the corner of the inner growth zone, in the position that is closest to the corresponding interaction are corner, here between the reservoirs B and C for CP_BC_. The red dashed line indicates the radius of the solid red line circle segment. Darker shades below the insert are trapped air bubbles. (D) Quantification of antibiotic interactions expressed as FICi for the image in panel C. The dotted line indicates a theoretical additive interaction with FICi = 1. Numerical values for this figure are available in S1 Data. CP, combination inhibitory point; FICi, fractional inhibitory concentration index; IC, inhibitory concentration; MEC, mecillinam; NIT, nitrofurantoin; TMP, trimethoprim; UTI, urinary tract infection.

To implement a specific synergy test, the prepared insert is placed into a culture plate and overcast with a final layer of culture agar. This step activates the assay, which is ready for use once the agar solidifies (Fig 1C). The final agar layer allows the antibiotics suspended in agar and housed in the reservoirs to start diffusing into the surrounding agar and toward the agar surface (Fig 1C). The isolate is then spread across the entire solidified agar to create a lawn by streaking with a cotton swab. The assay is designed for an inoculum density of 0.5 McFarland, in accordance with the European Committee on Antimicrobial Susceptibility Testing (EUCAST) guidelines for disk diffusion tests (version 8.0). Following inoculation, plates are incubated overnight to allow for sample growth. At this point, the CombiANT plates are identical to standard agar plates. CombiANT assays may thus be easily integrated into automated clinical systems for overnight culture and incubation of bacterial samples on plates. During growth, inhibition zones establish around the insert according to the diffusion-generated concentration landscape of the 3 antibiotics (Fig 2). For measurement of antibiotic interactions, the plates incubated overnight were imaged (e.g., with a hand-held mobile device or a gel-doc camera) and analyzed with an in-house curated algorithm that provided quantitative synergy measurements.

### Quantitative measurements of drug interactions

The insert is designed for easy and precise image analysis. Specifically, the geometry of the insert and its geometric relation to—i.e., its precise and predetermined distance from—the agar surface generates a predetermined and controlled diffusion of the antibiotics. The controlled diffusion is modeled with a finite elements method (FEM) for each antibiotic individually (S1 Text), yielding an antibiotic-specific diffusion map. The diffusion map expresses the concentration of the antibiotic for the surface of the plate relative to the initial concentration of the antibiotic in the reservoir and the diffusion coefficient of the antibiotic (Fig 2A).

Antibiotics differ in their diffusion rate due to their structure and the interaction with the diffusion matrix, in this case agar. The diffusion of an antibiotic is specific to the precise assay conditions (type of agar, culture volume, incubation time) used. Therefore, an experimental diffusion coefficient was pre-calculated for every antibiotic in an assay calibration step. To carry out the calibration of the assay, a reference strain with known MIC is tested with 3 concentrations of the target antibiotic (10 ×, 20 ×, 40 × MIC; see S1 Text for a detailed protocol). The calibration needs to be performed only once for every antibiotic, after which the diffusion map generated is stored and applied whenever the antibiotic is tested in subsequent assays. In addition to the diffusion coefficient generated, the recommended initial concentration of antibiotic to be supplemented with agar housed within the reservoirs is calculated from the one-time calibration result. The recommended initial concentration for the 8 antibiotics used in this study, according to EUCAST guidelines for antimicrobial susceptibility testing, are provided in S1 and S2 Tables.

The analysis algorithm uses the calibrated diffusion maps to generate a virtual antibiotic landscape to visualize the antibiotic concentrations in all different positions at the agar surface. First, the user indicates which antibiotic was placed in every reservoir of the assay. At this point, the algorithm recalls the stored diffusion maps corresponding to the antibiotics used in the assay and assembles them into an assay-specific antibiotic landscape (Fig 2B). The antibiotic landscape is then aligned to the assay picture, according to the geometric anchor points of the insert. The synergy quantification of CombiANT is based on the Loewe model for dose additivity [32]. The extent of antibiotic interaction is quantified from points on the edge of the inhibition zone (i.e., the concentration isobole of complete growth inhibition), according to the formula of the FICi, as specified below.

In an image of a CombiANT agar plate, certain areas of interest can be observed (Fig 2C). On the outside of the inserts in the area to the center of the reservoirs, every antibiotic is acting individually (the rest have negligible concentrations, see S3 Table). Since antibiotics also diffuse outward from the reservoirs, the point of the inhibition zone, opposite a chamber, that is farthest from the chamber represents the inhibitory concentration (IC) of that antibiotic when acting alone. For analysis, IC points are placed opposite the reservoir midpoint (Fig 2C). The IC points (shown in blue crosses in Fig 2C) are matched to the antibiotic landscape, and the corresponding concentrations of the 3 antibiotics are extracted (IC_A_, IC_B_, and IC_C_).

Inside the interaction imaging area (Fig 2C, black triangle), the 3 antibiotics have diffused out of the reservoirs and are now overlapping in pairs in the 3 corners. Every corner of the interaction imaging area constitutes a part of the plate where the 2 closest antibiotics are acting together. Therefore, the edge of the growth zone in the interaction imaging area that is closest to a corner corresponds to a point where the combination of the 2 antibiotics present is inhibitory to growth (shown in red points on Fig 2C). Similarly, to the 3 IC points, the 3 combination inhibitory points (CPs) are matched to points in the antibiotic landscape, and the concentrations of both antibiotics present are extracted. Having extracted both individual ICs and combinatorial ICs for all 3 antibiotics, the analysis algorithm proceeds to calculate FICi for all antibiotic pairs. For the interaction between antibiotics A and B, ${FICi}_{AB}=\frac{C_{A}}{{IC}_{A}}+\frac{C_{B}}{{IC}_{B}}$ in which *C_A_* and *C_B_* are the concentrations of A and B, respectively, in their corresponding CP. *FICi_AC_* and *FICi_BC_* are calculated similarly. An FICi value of 1 denotes additivity. FICi < 1 indicates synergy, whereas FICi > 1 indicates antagonism. Threshold values for clinically relevant levels of synergy and antagonism are usually set at <0.5, and >2–4, respectively [33–35]. The identification of all IC and CP points in an image can be done either automatically or manually. The analysis yields instantaneously the FICi values for all 3 antibiotic pairs (as shown in Fig 2D from the analysis of the plate in Fig 2C).

### Technical validation of assay

**Fig 3:**
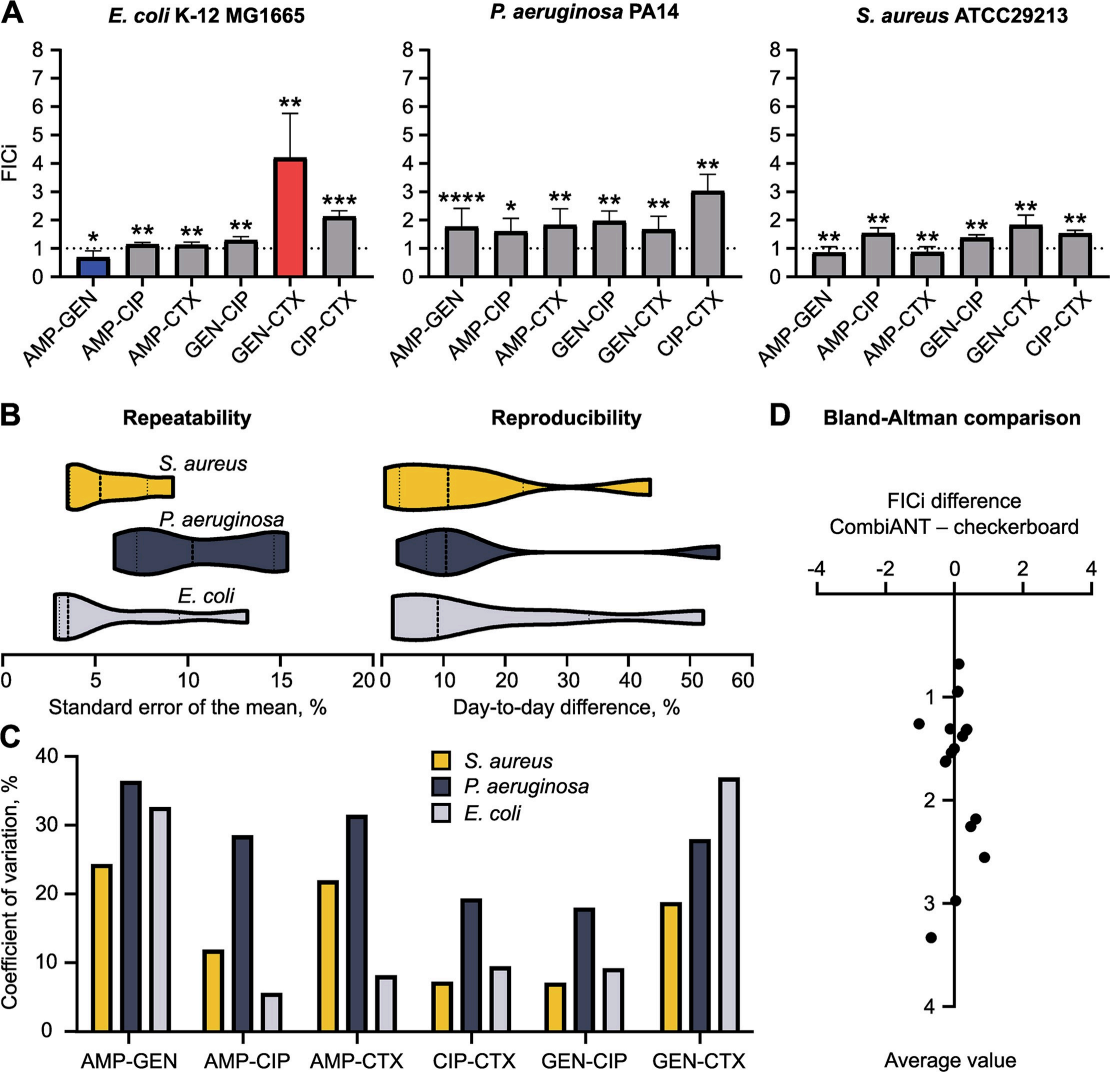
Technical validation of the CombiANT assay. (A) Quantification of drug interactions in reference strains of *E*. *coli*, *P*. *aeruginosa*, and *S*. *aureus*. Synergy is expressed by FICis (mean ± SD, *n* = 10 replicates). (B) Analysis of assay precision depicted in violin plots. Repeatability is expressed as relative standard error for assays conducted on the same day; dashed lines represent median standard error, and dotted lines represent the upper and lower quartiles, *n* = 6 combinations. Reproducibility is expressed as relative differences in FICi between days; dashed lines represent median relative differences, and dotted lines represent the upper and lower quartiles, *n* = 6 combinations. (C) Coefficient of variation for the pooled data (*n* = 10 replicates) encompassing both different-day and same-day replicates to quantify biological and technical replicability. Results are grouped by antibiotic interaction and then further subdivided between the 3 strains. (D) Bland-Altman method comparison of CombiANT and checkerboard assays (performed in house, mean values, *n* = 18 strain–antibiotic pair combinations). Absolute difference of FICi of methods is plotted against their average values. The small scattering of the points is indicative of little bias between the 2 methods. One sample Wilcoxon signed rank test against FICi = 1, ****P < 0.0001, ***P < 0.001, **P < 0.01, *P < 0.05. Numerical values for this figure are available in S1 Data. AMP, ampicillin; CIP, ciprofloxacin; CTX, cefotaxime; FICi, fractional inhibitory concentration index; GEN, gentamicin; SD, standard deviation.

First, as controls, we performed self-interaction experiments in which the 3 CombiANT insert reservoirs were filled with the same antibiotic. The self-interaction control experiments reliably produced FICi close to 1 for all antibiotics used in this study (S2 Fig). Next, to verify that the CombiANT assay produces valid synergy quantifications, we ran an accuracy and precision study. We tested all pairwise antibiotic interactions with 2 gram-negative reference strains (*E*. *coli* K-12 MG1665 and *P*. *aeruginosa* PA14) and a gram-positive reference strain (*S. aureus* ATCC29213) against a panel of 4 antibiotics. The antibiotics, ampicillin (AMP), cefotaxime (CTX), ciprofloxacin (CIP), and gentamicin (GEN), span 3 distinct mechanisms of action, and 2 are commonly used in treatments of bacteremia and sepsis caused by the bacterial species described. FICis were calculated for all pairwise interactions and the 3 strains (Fig 3A). To gage the model’s experimental validity, we extracted ICs for the antibiotics acting alone, i.e., MIC values, from the computational pipeline and compared these to MIC values from standard broth microdilution. The MIC values computed by the pipeline showed good agreement with those obtained from broth microdilution (S1 Fig). An extraction of MIC values from CombiANT assumes that the concentrations of the non-focal antibiotics are negligibly low at the IC points. We therefore calculated the expected concentrations of focal and non-focal antibiotics in the IC and CP points and found this assumption to be accurate (S3 Table). The highest concentration of non-focal antibiotic was approximately 2% of MIC.

In order to fully quantify the assay’s precision, the previously mentioned strains were screened in multiple replicates (*n* > 10) using the CombiANT assay protocol (S2 Text). Half of the replicates were tested on the same day to quantify repeatability (within-day variability). The other half was tested the following day to quantify reproducibility (day-to-day variability). To compare results between days, we chose to quantify relative day-to-day difference of the same antibiotic combination and then average all 6 combinations together for every strain (Fig 3B). Average relative differences of FICi measurements between days were below 13% for all strains, with the highest quartile being below 40%, indicating high reproducibility (Fig 3B, right side). To quantify repeatability, we measured the relative standard error of the mean for all FICi measurement, using same-day replicates for all antibiotic combinations. Those errors were then averaged for every strain. All relative standard errors were below 12%, with the highest quartile below 15%, indicating very small technical variation between replicates (Fig 3B, left side). Finally, we analyzed all replicates together to get an overall assessment of precision that encompasses both repeatability and reproducibility. We chose to calculate the coefficient of variation for each species-combination pair, an analysis that expresses the variability of measurement in proportion to an interaction’s average value (Fig 3C). All coefficients of variation were below 37% and on average 19.7%, signifying that the method was precise enough to be replicable and repeatable.

Next, we set out to quantify CombiANT’s accuracy. To that end, we replicated all measurements of antibiotic interactions using the gold standard methodology of broth-based checkerboard assays as previously described [27]. First, we tested whether the 2 methods (CombiANT and checkerboard assays) systematically produced different results. We therefore performed a Bland-Altman analysis of the FICi data that were obtained with the 2 methods (Fig 3D). The Bland-Altman comparison yielded a bias of 0.049 between the checkerboard and CombiANT assays. This low level of bias is close to the detection limit of FICi differences. Two FICi measurements with a difference of 0.049 would stem from 2 different CPs that would almost completely overlap. In CombiANT’s current dimension of 42 mm (diameter of insert) and with the resolution of a standard camera, that kind of position differences of the CPs is beyond the assay’s current resolution. Hence, we concluded that there is no statistically detectable discrepancy between results obtained from the 2 assays.

Having shown that the 2 methods are interchangeable, we tested the effect choosing one method over the other by performing a multivariate linear regression analysis. We tested the correlation between 2 factors and the outcome of the experiment in the form of FICi values. The 2 factors were the identity of the antibiotics used, and the method applied to that specific test (either CombiANT or checkerboard). The analysis yielded a correlation coefficient and a corresponding *P* value for every factor. As expected, the identity of the focal antibiotic pair had a strong and significant effect on the FICi value measured (with a correlation coefficient = 0.55; *P* < 0.01). The choice of method on the other hand was not statistically correlated with experimental outcome (correlation coefficient = 0.07; *P* = 0.83). Altogether, we concluded that the CombiANT assay has an equal accuracy for the detection of antibiotic interactions as broth-based checkerboard assays.

A difference between the CombiANT and checkerboard assays is that, by virtue of diffusion, CombiANT applies a continuous concentration range, while checkerboard assays typically test discrete 2-fold dilutions. Therefore, we tested whether the high precision of the CombiANT assay was a result of the continuous concentration range. To do that, we performed refined checkerboard assays with increased linear concentration ranges. All 18 strain-combination pairs, used in this technical validation, were replicated with this method and quantified using a Bliss independence null model (S3 Fig). The Bliss model was preferred because the analysis was easier computationally. Again, we observed high agreement of antibiotic interactions with CombiANT results (S3 Fig). Interestingly, synergy profiles occasionally showed dose-dependent variation, with different synergy profiles at low doses than at MIC, making interactions harder to classify (S3 Fig). Such dose-dependent variation was not detected by the CombiANT assay as it quantifies interactions at a predetermined high inhibition level. We concluded that the precision of the CombiANT assay is not just an effect of using a continuous concentration range.

### Antibiotic interaction panel using CombiANT assays on clinical UTI *E*. *coli* isolates

**Fig 4:**
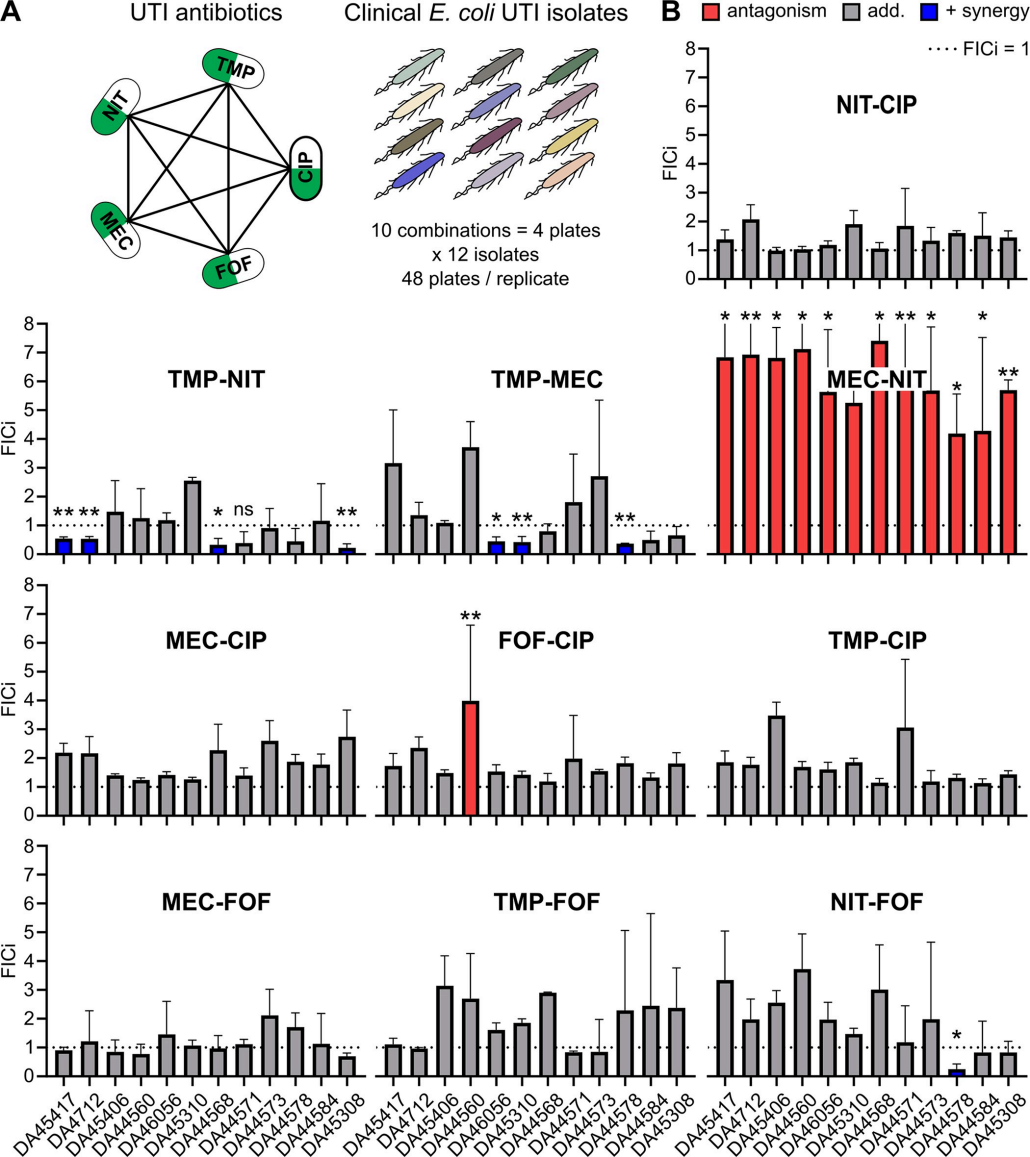
Interaction screening of *E*. *coli* UTI clinical isolates. (A) Schematic representation of the screening of 12 clinical isolates against a panel of 5 antibiotics. Ten pairwise interactions were quantified in every strain in *n* > 3 biological replicates, of which at least one was on a different day. (B) Antibiotic interactions expressed by FICi (mean ± SD). Clinically relevant levels of synergy (FICi < 0.5) are indicated in blue, while clinically relevant levels of antagonism (FICi > 4) are indicated in red. The upper measurement range in the screening was FICi = 8, which was exceed by several replicates and the MEC-NIT combination. These replicates were plotted as FICi = 8, and error bars that exceed this limit are trimmed. One-sample Wilcoxon signed rank test against FICi = 1, ***P < 0.001, **P < 0.01, *P < 0.05. Numerical values for this figure are available in S1 Data. CIP, ciprofloxacin; FICi, fractional inhibitory concentration index; FOF, fosfomycin; MEC, mecillinam; NIT, nitrofurantoin; SD, standard deviation; TMP, trimethoprim; UTI, urinary tract infection.

We proceeded to use CombiANT assays to screen for antibiotic synergies against 12 different *E*. *coli* UTI clinical isolates and included the *E*. *coli* K-12 MG1655 as a reference strain (S4 Fig). Here, a panel of 5 antibiotics that are commonly used as single or combination treatment for UTIs was selected [36,37], nitrofurantoin (NIT), trimethoprim (TMP), mecillinam (MEC), CIP, and fosfomycin (FOF). CombiANT assays were used to measure all pairwise interactions of the antibiotics panel against the 12 *E*. *coli* strains (Fig 4A). The *E*. *coli* strains were designated susceptible to all antibiotics in the panel. The categorical FICi limit for an interaction to be designated to show clinically relevant levels of positive synergy was set at FICi < 0.5, according to previous recommendations [33–35]. A conservative limit for antagonism was set at FICi > 4 [34,35]. All in-between values were designated as describing additivity.

Overall, a majority of the combinations were shown to be additive in nature, with NIT-CIP, MEC-CIP, TMP-CIP, MEC-FOF, TMP-FOF, and combinations being additive across all tested strains. FOF-CIP exhibited a borderline but statistically significant antagonistic interaction for one isolate (DA44560) but was observed to have an additive behavior in all remaining strains. In contrast, NIT-FOF had a slight but significant positive interaction in the strain DA44578 but was additive in all other strains. The medically more interesting results were obtained for the remaining 3 combinations, TMP-NIT, TMP-MEC, and MEC-NIT. Positive synergy for TMP-NIT was detected in only 4 out of 12 UTI isolates (TMP-NIT: DA45417, DA4712, DA44568, and DA45308). TMP-MEC exhibited positive synergy in 3 out of the 12 isolates (TMP-MEC: DA46056, DA45310, and DA44578). All remaining isolates in both cases displayed no synergy between the antibiotics, indicating genetic variation in the nature of these interactions among UTI isolates. For the clinically relevant combination of TMP-MEC strains DA44560, DA45406, and DA46056 exhibited entirely different synergy profiles, ranging from border line antagonism to positive synergy. We confirmed further the variation in TMP-MEC synergy by performing additional synergy experiments on those strains using a time-kill assay and a growth-rate assay. Both assays reiterated the findings of CombiANT (S5 and S6 Figs). Finally, the combination MEC-NIT exhibited a strongly antagonistic behavior that was detected on all of the tested strains. Together, these data clearly indicate a value of case-by-case synergy validation within one species.

## Discussion

In this study, we present and characterize the new CombiANT assay, which enables an efficient determination of antibiotic interactions. Our extensive technical validation indicated high accuracy and precision, and an overall equal performance to the established checkerboard assay. We then implemented CombiANT for a screen of antibiotic synergy among UTI isolates. A consistent antagonistic interaction was discovered as well as 4 interactions with significant strain-to-strain variation, indicating the potential of CombiANT for both individualized clinical diagnostics and basic research.

An alternative methodology has been successfully applied for the high-throughput measurement of drug interactions in basic research [26]. In this method, bacteria are treated with low sub-MIC concentrations of different antibiotics and combinations thereof, and their exponential growth rate recorded over time using absorbance. While these measurements are highly precise, they are less applicable for clinical microbiology, requiring dedicated instrumentation and complex data analyses. Therefore, the CombiANT assay was specifically designed for simplicity and practical implementation in clinical settings. In the inactive state, inserts can be kept refrigerated, in large amounts, according to the regular shelf-life of antibiotic agar plates. This makes it feasible for hospitals and laboratories to pre-load inserts with the desired antibiotics of interest and then quickly implement them when needed. Starting with the activation step, the handling of CombiANT plates is identical to that of regular agar plates and thus compatible with the existing clinical pipelines for the mass handling of agar plates, including automated pouring of agar for large-scale production.

To allow for easy use of CombiANT both by clinics and academic laboratories, we designed 2 different protocols, namely (i) a resistance breakpoint-based protocol for clinical use (S2 Table, and as applied for the UTI screen in Fig 4), and (ii) a higher-sensitivity MIC-based protocol for research applications (S1 Table, as applied for Fig 3). CombiANT can be fully automated and requires a digital picture as input for image analysis. The lack of need for dedicated machinery makes CombiANT suitable also for low-resource settings.

An important design principle of CombiANT was that antibiotic synergy is quantified at high, clinically relevant concentrations. Synergy is measured from the edge of inhibition zones, meaning the MIC-equipotency isobole of combination space using the FICi. The FICi method is based on the null model of Loewe additivity of doses [32]. An alternative approach for synergy quantification uses the Bliss independence null model that assumes additivity of effects [32], rather than doses. The general direction of antibiotic interactions is often conserved between Bliss and Loewe models, although exact agreement exists in only few dose-response points. Synergy can be measured at lower antibiotic concentration and inhibition levels than used in CombiANT, using, e.g., growth rates as readout. It has been shown previously that interaction profile of a particular antibiotic combination can be dose dependent [38–40], occasionally complicating synergy quantification from checkerboards assays (S3 Fig). These biologically interesting cases suggests complex physiological effects. Synergy measurements by FICi are robust (or blind) to such variation, as they are performed at a high inhibition level (MIC), which we would suggest is more clinically relevant. Another technical difference of the plate-based CombiANT assay compared to broth microdilution methods refers to the phenotypic effects of antibiotics on cell shape. Many antibiotics induce changes in cell shape, as part of their mechanism of action. For example, beta-lactam antibiotics that inhibit PBP3 and septation induce extensive cell elongation prior to cell death [41]. Such elongation can lead to an overestimation of viable cell numbers by optical density measurements, leading, e.g., to a disagreement of MIC values called by broth and agar methods [42].

The results obtained in the datasets in Figs 3 and 4 agree with those described in the literature. CombiANT replicated the previously reported synergies between AMP-GEN, TMP-MEC, and TMP-NIT combinations; the strong antagonism between MEC and NIT; and the additivity between beta-lactams and CIP in *E*. *coli* K-12 MG1655 [26,43]. However, the additive combination GEN-CIP was previously classified as synergistic using the low-inhibition growth rate methodology [43]. Only a few antibiotic interactions in *P*. *aeruginosa* PA14 strain and the *S*. *aureus* ATCC29213 were previously characterized, limiting comparisons to our study. AMP-CIP additivity was previously reported for *S*. *aureus* [44], and interactions of CIP with beta-lactams and CIP with the aminoglycoside GEN are known to be antagonistic in *P*. *aeruginosa* [18], and CombiANT replicated these observations. Taken together, the observed high agreement of our measurements with the literature support the accuracy and utility of CombiANT.

The screening of 12 UTI clinical isolates presented in Fig 4 illustrates CombiANT’s usability for clinical microbiology. To mimic a clinical scenario, we had no specific inclusion criteria for these isolates, aside from requiring susceptibility according to EUCAST breakpoint. The applied CombiANT protocol variant with breakpoint-based input concentrations (S2 Table) reliably produced readable growth zones in the isolates, requiring no isolate-specific adjustments. However, some of the detected antagonism was so strong that growth did occur up to the reservoir edge and thereby exceeded the upper quantification limit of FICi = 8 in the screen. Resistant strains were not included in this analysis. The reason for that was 2-fold. First, we wanted to illustrate that—even in isolates that, in a clinical perspective, are viewed as being similar (since they are all deemed susceptible)—antibiotic interaction profiles can vary considerably from isolate to isolate. Second if resistance is detected in one of the antibiotics that could potentially go into a multitherapy scheme, the logical first choice would be to replace that antibiotic with another one to which the strain is susceptible. The behavior and utility of CombiANT with resistant and multi-resistant isolates is an important future research direction.

As the above synergy screening of 12 clinical UTI isolates iterates, there are cases, such as the interaction between TMP and MEC, in which combining 2 antibiotics seems to have a consistent antagonistic effect across most strains. Cases such as these, with antagonistic behavior across the board, illustrate the need for clear guidance when designing combination therapies, even when carried out empirically. Identifying such combinations of antibiotics that should be avoided will require large-scale systematic synergy screenings. With the current methods that quantify antibiotic synergy, a systematic screening of such a scale is unfeasible. However, CombiANT presents a new, less labor-costly method that is still capable of quantifiable results. Using our new approach to interaction studies makes such large-scale synergy screens attainable. The screening performed here illustrates CombiANT’s scalability. To get all pairwise interactions for the 5 antibiotics that we screened one UTI isolate for and assuming that a range of 8 concentrations is tested for all antibiotics would require 640 tubes for a time-kill experiment or ten 96-well plates for a checkerboard assay. With CombiANT, all synergy data were extracted from only 4 agar plates.

The screening for the interactions between TMP-NIT and TMP-MEC revealed an important result, namely that the same 2 antibiotics might not have a consistent synergy profile across different strains of the same species. If antibiotics can be synergistic against one strain but additive or antagonistic for another, then we suggest that synergy screens should become a part of standard testing in clinical microbiology laboratories. Thus, an assay such as CombiANT, which is quantitative and simple, could be part of routine screening in a microbiology laboratory. Translating CombiANT’s in vitro positive synergy findings to in vivo antibiotic synergy is not trivial. Antibiotic absorption as well as kinetics in the human body might disrupt a positive synergy profile. In silica antagonistic behavior, however, represents a compound-to-compound inhibition in its simplest form. Therefore, we think any detection of antibiotic antagonism in vitro could be a helpful guide for clinical decisions in an even more straightforward way than antibiotic synergy.

Aside from the clinical applicability, CombiANT has high potential as a tool for basic research in biology. For a majority of observed antibiotic interactions, a mechanistic and evolutionary understanding is currently lacking, and these knowledge gaps may partly be explained by the complexity of current synergy measurement methods. In our limited screen of UTI isolates, the majority of antibiotic interactions were additive. The variable synergy profiles that were observed with TMP-NIT, FOF-CIP, NIT-FOF, and TMP-MEC indicate interesting biological strain-to-strain variation that warrants further investigation. The highly antagonistic interaction between the beta-lactam MEC and NIT may potentially indicate an evolutionary conservation of this drug interaction, implying a functional constraint between cellular functional modules. The antagonism could potentially be explained by an overlap in the cellular drug and stress responses to the component drugs. It has been shown that both beta-lactams and NIT individually induce expression of the cellular SOS response for DNA repair [45,46], potentially explaining the antagonism through a coordinated stronger defense response. Yet the antibiotics also induce other response systems, such as the RpoS-mediated stress response for beta-lactams [47] and oxidative stress response in the case of NIT [48]. Therefore, the antagonism observed could alternatively be explained by potential pleiotropy of these other responses. These and other hypotheses could be efficiently tested by implementing CombiANT for a functional genetics screen.

Finally, CombiANT is not restricted to the study of interactions between antibiotics. In principle, the individual and combined activity of any bioactive compound can be studied with CombiANT. We anticipate similar applications in cell biology, as well as environmental microbiology. A potential diversity of applications is supported by the high flexibility of CombiANT, which can be easily implemented with different growth medium and culturing conditions (e.g., temperature). We anticipate that CombiANT could, e.g., be applied for the characterization of combination effects between chemotherapy agents on hydrogel-embedded growth of spheroids or organoids [49,50]. Thus, CombiANT may be an interesting alternative to the currently utilized microfluidic approaches [51] that in our experience [52–54] entail high assay complexity. CombiANT could also be applied to characterize biological activities, independent of several inducers and repressors, provided that the activity can be optically tracked (e.g., fluorescence, colorimetry). Still, we currently see the highest need for efficient interaction testing with antibiotic synergies. The spread of antibiotic resistance increasingly makes combination therapy attractive, but the high pathogen diversity indicates an added value of personalized synergy validation and optimization, even for isolates that appear broadly resistant.

## Materials and methods

### Fabrication and design

The insert was designed with computer-aided design software (Autodesk Fusion 360, Adobe) and manufactured by 3D printing (Formlabs, Somerville, MA; SLA 3D printer) using proprietary formulations for autoclavable or dental resin version 2 (Formlabs, Somerville, MA; SLA 3D printer). The 3D printing was performed in the U-Print facility of Uppsala University. The designs are available upon request to the authors.

### Strains and growth media

For the technical validation, we tested the reference strains *E*. *coli* K-12 MG1655 (DA5438), *P*. *aeruginosa* PA14 (DA64160), and *S*. *aureus* ATCC29213 (DA64485). For the UTI study, we screened 12 clinical isolates of independent origin that were susceptible to the antibiotics CIP, FOF, MEC, NIT, and TMP. Bacteria were cultured on Mueller-Hinton agar and in Mueller-Hinton broth (Becton Dickinson, Sparks, MD; Refs. 275730, 225250) with incubations at 37°C. Overnight cultures were prepared from single colonies in 1 mL and 190 rpm orbital shaking. For the UTI isolate screening, the agar was supplemented with 25 mg/L glucose-6-phosphate, as this is required for the action of FOF. Antibiotic stocks were prepared according to the manufacturer’s recommendations and stored frozen at −20°C in aliquots for single use: AMP 100 mg/mL in water (Sigma-Aldrich, Ref. A9518-25G), CIP 25 mg/mL in 0.1 M HCl (Sigma-Aldrich, Ref. 17850-25G-F), CTX 50 mg/mL in water (Sigma, Ref. C7039-1G), GEN 50 mg/mL in water (Sigma, Ref. 48760-5G-F), FOF 50 mg/mL in water (Sigma-Aldrich, Ref. P5396-5G), MEC 10 mg/mL in water (Sigma-Aldrich, Ref. 33447-100MG), NIT 10 mg/mL in 100% DMSO (Sigma, Ref. N7878-10G), and TMP 10 mg/mL in 100% DMSO (Sigma, Ref. T-7883-5G).

### Broth microdilution

To calibrate CombiANT, we determined MIC values for antibiotics individually, using standard broth microdilution methodology in agreement with EUCAST guidelines. Two-fold serial dilutions of antibiotics in Mueller Hinton broth were prepared in 96-well microtiter plates. The plates were then inoculated with approximately 3 × 10^5^ cells from a dense overnight culture (1:1000 dilution, 180 μL final volume) and incubated without shaking at 37°C for 24 hours, after which wells were mixed by pipetting and growth was measured by optical density at 540 nm (Thermo Fisher Scientific, Multiscan FC Type 357). MIC was called at the lowest concentration that yielded a growth signal of uninoculated control wells. Measurements were performed with 2 biological replicates, and their average value was designated MIC. For determination of MIC to FOF, the media were supplemented with 25 mg/L of glucose-6-phosphate, which is required for FOF-mediated inhibition.

### Input concentrations

For the technical calibration of the CombiANT assay, the antibiotic concentrations were determined based on the MIC of the antibiotic against the strains used (S1 Table). In the screening of the UTI isolates, a set concentration of antibiotic was used for all strains (S2 Table). When MIC is known for an antibiotic, using a MIC-based determination of the insert’s concentration is preferable, as it will lead to the most readable results. For tests with FOF, glucose-6-phosphate was provided in the final agar layer at a concentration of 25 mg/L.

### Checkerboard experiments

Standard checkerboard assays (8 × 8 concentrations) for FICi determination were performed in single biological replicate and with 2-fold serial dilutions ranging from 4 × MIC to one-quarter MIC, as previously described [27]. Inoculum size was 5 × 10^5^ cells (0.5 McFarland), and optical density was read at 540 nm (Thermo Fisher Scientific, Multiscan FC Type 357) after 16 hours of static incubation. For Bliss model synergy quantification, higher-resolution checkerboards (9 × 9 concentrations) were obtained using linear concentrations up to 1 × MIC, and with 3 biological replicates. Treatments positions were fully randomized to avoid bias from edge and gradient effects. Degree of synergy was calculated as previously described [55]. Growth yield was expressed relative to untreated wells using background-corrected optical density values. The expected relative growth *Y*_*1+2*_ according to a Bliss independence model [56] was calculated by multiplication of the relative growth yields *Y*_*1*_ and *Y*_*2*_ obtained in the single-antibiotic treatments. The degree of synergy *S* of a combination is defined as: *S = Y*_*1+2*_ *–Y*_*observed*_. *S = 0* expresses additivity, positive values denote synergy, and negative values denote antagonism.

### Physics diffusion model and image analysis

An FEM was used to model diffusion of reagents from the reservoirs. Diffusion was assumed to follow Fick’s laws of diffusion, and concentration of the agent was calculated by solving the convection-diffusion equation for no advective flux and no net volumetric source. The diffusion coefficients used were experimentally determined for every antibiotic. As per the protocol described in the Supporting Information (S1 Text), each experimental coefficient is unique to the specific temperature, medium, and antibiotic used. The FEM analysis, antibiotic diffusion modeling, and calibration as well as the antibiotic landscape assembly were performed using COMSOL Multiphysics (Comsol, Stockholm, Sweden). The algorithm was scripted using Matlab (Mathworks, Natick, MA) and COMSOL-Matlab bridge and is provided as S1 Code. CombiANT plates were photographed using a vertically mounted CCD digital camera (Raspberry Pi version 2 camera module). The IC and CP points were identified manually according to these principles. IC points were placed on the inhibition edge outside the interaction imaging area and opposite the midpoint of the corresponding reservoir. The CP point is selected within the interaction imaging area as the point of the growth zone that is closest to the corresponding corner of the interaction imaging area. The coordinates of the IC and CP points were obtained from the image using the multi-point tool of the ImageJ distribution Fiji. The coordinates of the interaction imaging area were obtained in the same way and used to align the image with the concentration landscape. The computation of ICs and FICi is automated, and the analysis script is provided as S1 Code. To avoid observer bias, the manual identification of the IC and CP points was performed blind to the strains and antibiotics used on the plate. Experiments were analyzed by a single individual to exclude potential person-to-person bias.

### Statistical analyses

Statistical analyses were performed using Graph Pad Prism and Matlab. Statistical difference of measured FICi to the additive model (FICi = 1) was assessed using Wilcoxon signed rank tests. In the UTI screen, only interactions that show clinically relevant levels of antibiotic interaction (FICi < 0.5, FICi > 4) were tested. Numerical values underlying all analyses are available in S1 Data.

### Time-kill experiments

A time-kill experiment was performed with *E*. *coli* isolates DA44560, DA45406, and DA46056, which showed different interaction profiles for the combination MEC-TMP. In brief, MHB broth was supplemented with TMP, MEC at isolate-adjusted MIC dosage, or the MEC-TMP combination (MIC-MIC), of which 1 mL was inoculated with approximately 3 × 10^6^ cells from a dense overnight culture (1:1000 dilution). The MICs of the isolates were determined before the assay using broth microdilution and were found to vary between 0.1 to 0.4 mg/L for both antibiotics. Cultures were incubated at 37°C with orbital shaking, and survival was measured after 0, 2, 4, 8, and 24 hours by plating diluted samples on well-dried MHA using Miles and Misra spotting [57] with 5 μL droplets. Colony forming units (CFU) were counted after overnight incubation, and stability of counts was verified using extended incubation up to 48 hours. Experiment and counting were done without knowing the condition identities (blind). The experiment was performed with 3 biological replicates. Antibiotic synergy was assessed by comparison with a Bliss null model, for which the expected additive combination effects was obtained by multiplication of the survival fractions of the antibiotics acting alone.

### Growth curve experiments

Growth curves of *E*. *coli* isolates DA44560, DA45406, and DA46056 were obtained for a 5 × 5 concentration checkerboard of MEC-TMP and measurement of growth using optical density at 600 nm (OD) in a Bioscreen C plate reader (OY Growth Curves, Helsinki, Finland; Ref. FP-1100-C). The strains were selected for their variation in synergy profiles for this antibiotic combination. Antibiotics were dosed at 0, 0.025, 0.05, 0.1, and 0.2 mg/L. Treatment conditions were prepared in large volume of which 300-μL portions were aliquoted into honeycomb well plates. To start antibiotic treatments, the wells were inoculated with approximately 3 × 10^6^ cells from a dense overnight culture. Immediately after inoculation, plates were transferred to Bioscreen reader for incubation (37°C, orbital shaking with medium amplitude and normal speed) and measurements of OD every 4 minutes for a total of 12 hours. The experiment was performed with 3 biological replicates. Data were analysed using the *R* statistical platform. Raw OD values were background corrected using OD from uninoculated wells. Exponential growth rates were calculated from log-transformed data (ln OD) in the early time window of 60 minutes to 110 minutes during which growth was strictly exponential (the fit of an exponential model showed a median coefficient of determination *R*^*2*^ of 0.99, and a range of 0.97–1.0; see S6 Fig). Relative growth rate inhibition effects *E* were calculated as 1 –(*R*_*xy*_−*R*_*0*_), in which *R*_*0*_ represents the uninhibited growth of untreated reference wells. The expected inhibition effect *E*_*1+2*_ was calculated by addition of the inhibition effects of the antibiotics when acting alone, *E*_*1*_ and *E*_*2*_. Synergy was then quantified, using degree of synergy *S*, expressed as *S = E*_*1+2*_ *–E*_*observed*_. Positive values denote synergy, and negative values denote antagonism. The obtained S-values were then averaged across the checkerboard concentration grid and tested against the null expectation (*S = 0*) using a one-sample Wilcoxon signed rank test. A parallel analysis was conducted using growth yields after 12 hours, according to the procedures described in the section ‘Checkerboard experiments,’ given earlier.

## Supporting information

S1 Fig Validity of calibrated physics models. Comparison of the MIC values obtained with CombiANT as part of the synergy calculations with those obtained from BMD. Data of reference strains *E*. *coli*, *P*. *aeruginosa*, and *S*. *aureus* tested against the antibiotics AMP, CIP, GEN, and CTX. CombiANT MIC predictions (indicated by solid points, mean ± SD, *n* = 18, 3 biological replicates of 6 technical replicates each) fall within the log2 dilution window of the BMD assay (indicated by the shaded regions), supporting the physics diffusion model. An accurate determination of MIC for *P*. *aeruginosa* by BMD was not possible for AMP and CTX due to extensive cell elongation preceding cell death. Numerical values for this figure are available in S1 Data. BMD, broth microdilution.
(TIF)

S2 Fig Self-interaction experiments of the *E*. *coli* reference strain K-12 MG1655. FICi for all self-interactions for the 8 antibiotics AMP, CIP, CTX, GEN, FOF, NIT, MEC, and TMP. Bars indicate average values and SD from 3 biological replicates. The dotted line indicates additivity, i.e., FICi = 1. All interactions were additive in nature and showed no statistically significant difference from FICi = 1 according to one-sample Wilcoxon signed rank test. Numerical values for this figure are available in S1 Data. FICi, fractional inhibitory concentration index
(TIF)

S3 Fig High-sensitivity checkerboard analysis using Bliss independence model. (A) Antibiotic checkerboard data with linear concentrations ranges for *E*. *coli*, *P*. *aeruginosa*, and *S*. *aureus* tested against the antibiotics AMP, CIP, GEN, and CTX. Each antibiotic was tested with 8 equally spaced linearly increasing concentrations (denoted 1–8) between zero and the MIC for the respective species. Data are average growth values of 3 biological replicates relative to untreated controls. (B) Dose-dependent synergy profiles according to a Bliss independence additive model. Numerical values for this figure are available in S1 Data.
(TIF)

S4 Fig CombiANT UTI antibiotic interactions for the *E*. *coli* reference strain K-12 MG1655. FICi for all pairwise combinations of the 5 antibiotics CIP, FOF, NIT, MEC, and TMP. Bars indicate average values and SD from 3 biological replicates. The dotted line indicates additivity, i.e., FICi = 1. Synergy with FICi < 0.5 is indicated in blue, antagonism with FICi > 4 is indicated in red. One-sample Wilcoxon signed rank test against FICi = 1, *P < 0.05. Numerical values for this figure are available in S1 Data. FICi, fractional inhibitory concentration index
(TIF)

S5 Fig Time-kill assay with MEC-TMP for 3 *E*. *coli* clinical isolates. Time-kill assay on the UTI strains DA44560, DA45406, and DA46056. The 3 strains showed contrasting interaction profiles for the MEC-TMP combination in the CombiANT assay, which was additive for DA45406, synergistic for DA46056, and mildly antagonistic for DA44560 (although FICi < 4). Survival fraction was expressed as CFU/mL relative to time point 0 hours (mean ± SD, *n* = 3 biological replicates). Antibiotics were applied at isolate-adjusted MIC dosage. The circle symbols (●) denote the effect of MEC on strain survival, triangles (π) denote the effect of TMP, and open squares (ο) denote the combined effect of the MEC-TMP combination. The red lines represent the Bliss additive interaction model (calculated as the multiplication of survival rates, of the antibiotics when acting individually). Increased survival in the combination compared to the additivity line indicates antagonism, whereas the opposite indicates synergy. The dotted line shows the assay’s limit of certainty. Missing data points indicate a colony count of zero (not defined for logarithmic scales). Numerical values for this figure are available in S1 Data. CFU, colony forming units
(TIF)

S6 Fig Growth rate assay with MEC-TMP for 3 *E*. *coli* clinical isolates. The UTI isolates DA44560, DA45406, and DA46056 were treated with low concentrations of MEC, TMP, or combinations of MEC-TMP, using a 5 × 5 checkerboard setup. The 3 strains showed contrasting interaction profiles for the MEC-TMP combination in the CombiANT assay, which was additive for DA45406, synergistic for DA46056, and mildly antagonistic for DA44560 (although FICi < 4). Growth was monitored in 4-minute intervals using OD. (A) Exponential growth rates of the checkerboard, and degree of synergy *S* in drug–drug combinations according to a Bliss additive model (growth rate inhibition values are added). Positive values of *S* indicate synergy, and negative values indicate antagonism. Values are averages of 3 biological replicates. (B) Distribution of coefficient of determination *R*^*2*^ for fitted exponential growth models in the measurement time window of 60 to 110 minutes after start of treatment. (C) Average degree of growth rate synergy across the concentration grid (mean ± SD, *n* = 16 combinations). (D) Growth yields after 12 hours of treatment, and calculated degree of synergy according to a Bliss additive model (relative yields are multiplied). Values are averages of 3 biological replicates. (E) Logarithmic presentation of underlying growth curves. The dotted lines show the measurement window for exponential growth rates in panel A. (F) Average degree of growth yield synergy across the concentration grid (mean ± SD, *n* = 16 combinations). MEC-TMP additivity in DA45406 is replicated by growth rate and growth yield measurements. MEC-TMP synergy in DA46056 is apparent in growth yields, but not growth rates. Mild antagonism of MEC-TMP in DA44560 is replicated in growth rates, but not growth yields. Numerical values for this figure are available in S1 Data. OD, optical density at 600 nm
(TIF)

S1 File Stereolithography file for the CombiANT insert. (STL)

S1 Code Analysis script for CombiANT. (DOCX)

S1 Table Antibiotic input concentrations for validation study in Fig 3. MIC-based initial concentrations for the technical calibration with reference strains *E*. *coli*, *P*. *aeruginosa*, and *S*. *aureus* and the antibiotics AMP, CIP, GEN, and CTX.
(PNG)

S2 Table Antibiotic input concentrations for the exploratory study in Fig 4. Breakpoint-based initial concentrations, as applied for the synergy screening of the *E*. *coli* UTI isolates.
(PNG)

S3 Table Antibiotic concentrations in IC and CP points. Concentrations of AMP, CIP, and GEN in a CombiANT plate of *E*. *coli* K-12 MG1665. Concentrations in every point are expressed as fold of MIC for that specific antibiotic in a broth microdilution.
(PNG)

S1 Text Calibration protocol. The calibration protocol for the use of new media-antibiotic combination in the CombiANT assay.
(DOCX)

S2 Text Protocol for CombiANT assay. The execution protocol for the CombiANT assay.
(DOCX)

S1 Data (XLSX)

## Abbreviations

AMP: ampicillin
CFU: colony forming units
CIP: ciprofloxacin
CP: combination inhibitory point
CTX: cefotaxime
EUCAST: European Committee on Antimicrobial Susceptibility Testing
FEM: finite elements method
FICi: fractional inhibitory concentration index
FOF: fosfomycin
GEN: gentamicin
IC: inhibitory concentration
MEC: mecillinam
MIC: minimal inhibitory concentration
NIT: nitrofurantoin
OD: optical density at 600 nm
SD: standard deviation
TMP: trimethoprim
UTI: urinary tract infection
VRSA: vancomycin-resistant Staphylococcus aureus

## References

[1] Kang C-I, Kim S-H, Park WB, Lee K-D, Kim H-B, Kim E-C, et al Bloodstream Infections Caused by Antibiotic-Resistant Gram-Negative Bacilli: Risk Factors for Mortality and Impact of Inappropriate Initial Antimicrobial Therapy on Outcome. Antimicrobial Agents and Chemotherapy. 2005;49: 760–766. 10.1128/AAC.49.2.760-766.2005 15673761 PMC547233

[2] Montravers P, Veber B, Auboyer C, Dupont H, Gauzit R, Korinek AM, et al Diagnostic and therapeutic management of nosocomial pneumonia in surgical patients: Results of the Eole study: Critical Care Medicine. 2002;30: 368–375. 10.1097/00003246-200202000-00017 11889312

[3] Tängdén T. Combination antibiotic therapy for multidrug-resistant Gram-negative bacteria. Upsala Journal of Medical Sciences. 2014;119: 149–153. 10.3109/03009734.2014.899279 24666223 PMC4034552

[4] Daikos GL, Tsaousi S, Tzouvelekis LS, Anyfantis I, Psichogiou M, Argyropoulou A, et al Carbapenemase-producing Klebsiella pneumoniae bloodstream infections: lowering mortality by antibiotic combination schemes and the role of carbapenems. Antimicrob Agents Chemother. 2014;58: 2322–2328. 10.1128/AAC.02166-13 24514083 PMC4023796

[5] Sewick A, Makani A, Wu C, O’Donnell J, Baldwin KD, Lee G-C. Does Dual Antibiotic Prophylaxis Better Prevent Surgical Site Infections in Total Joint Arthroplasty? Clin Orthop Relat Res. 2012;470: 2702–2707. 10.1007/s11999-012-2255-1 22290130 PMC3441989

[6] Marino K, Parlee A, Orlando R, Lerner L, Strymish J, Gupta K. Comparative Effectiveness of Single versus Combination Antibiotic Prophylaxis for Infections after Transrectal Prostate Biopsy. Antimicrob Agents Chemother. 2015;59: 7273–7275. 10.1128/AAC.01457-15 26369958 PMC4649242

[7] Nseir S, Favory R, Jozefowicz E, Decamps F, Dewavrin F, Brunin G, et al Antimicrobial treatment for ventilator-associated tracheobronchitis: a randomized controlled multicenter study. Crit Care. 2008;12: R62 10.1186/cc6890 18454864 PMC2481443

[8] Döring G, Flume P, Heijerman H, Elborn JS, Consensus Study Group. Treatment of lung infection in patients with cystic fibrosis: current and future strategies. J Cyst Fibros. 2012;11: 461–479. 10.1016/j.jcf.2012.10.004 23137712

[9] Nahid P, Dorman SE, Alipanah N, Barry PM, Brozek JL, Cattamanchi A, et al Executive Summary: Official American Thoracic Society/Centers for Disease Control and Prevention/Infectious Diseases Society of America Clinical Practice Guidelines: Treatment of Drug-Susceptible Tuberculosis. Clin Infect Dis. 2016;63: 853–867. 10.1093/cid/ciw566 27621353 PMC6366011

[10] Vestergaard M, Paulander W, Marvig RL, Clasen J, Jochumsen N, Molin S, et al Antibiotic combination therapy can select for broad-spectrum multidrug resistance in Pseudomonas aeruginosa. International Journal of Antimicrobial Agents. 2016;47: 48–55. 10.1016/j.ijantimicag.2015.09.014 26597931

[11] Tueffers L, Barbosa C, Bobis I, Schubert S, Höppner M, Rühlemann M, et al Pseudomonas aeruginosa populations in the cystic fibrosis lung lose susceptibility to newly applied β-lactams within 3 days. J Antimicrob Chemother. 2019;74: 2916–2925. 10.1093/jac/dkz297 31355848

[12] Bloemberg GV, Keller PM, Stucki D, Stuckia D, Trauner A, Borrell S, et al Acquired Resistance to Bedaquiline and Delamanid in Therapy for Tuberculosis. N Engl J Med. 2015;373: 1986–1988. 10.1056/NEJMc1505196 26559594 PMC4681277

[13] Worthington RJ, Melander C. Combination approaches to combat multidrug-resistant bacteria. Trends in Biotechnology. 2013;31: 177–184. 10.1016/j.tibtech.2012.12.006 23333434 PMC3594660

[14] Perichon B, Courvalin P. VanA-Type Vancomycin-Resistant Staphylococcus aureus. Antimicrobial Agents and Chemotherapy. 2009;53: 4580–4587. 10.1128/AAC.00346-09 19506057 PMC2772335

[15] Bush K. Beta-lactamase inhibitors from laboratory to clinic. Clinical Microbiology Reviews. 1988;1: 109–123. 10.1128/CMR.1.1.109 3060240 PMC358033

[16] Band VI, Hufnagel DA, Jaggavarapu S, Sherman EX, Wozniak JE, Satola SW, et al Antibiotic combinations that exploit heteroresistance to multiple drugs effectively control infection. Nature Microbiology. 2019; 1 10.1038/S4_1564-019-0480-z PMC7205309 31209306

[17] Chait R, Craney A, Kishony R. Antibiotic interactions that select against resistance. Nature. 2007;446: 668–671. 10.1038/nature05685 17410176

[18] Barbosa C, Beardmore R, Schulenburg H, Jansen G. Antibiotic combination efficacy (ACE) networks for a Pseudomonas aeruginosa model. PLoS Biol. 2018;16: e2004356 10.1371/journal.pbio.2004356 29708964 PMC5945231

[19] Dean Z, Maltas J, Wood K. Antibiotic interactions shape short-term evolution of resistance in E. faecalis. PLoS Pathog. 2020;16: e1008278 10.1371/journal.ppat.1008278 32119717 PMC7093004

[20] Plotz PH, Davis BD. Synergism between streptomycin and penicillin: a proposed mechanism. Science. 1962;135: 1067–1068. 10.1126/science.135.3508.1067 14487239

[21] Gonzales PR, Pesesky MW, Bouley R, Ballard A, Biddy BA, Suckow MA, et al Synergistic, collaterally sensitive β-lactam combinations suppress resistance in MRSA. Nat Chem Biol. 2015;11: 855–861. 10.1038/nchembio.1911 26368589 PMC4618095

[22] Bollenbach T, Quan S, Chait R, Kishony R. Nonoptimal Microbial Response to Antibiotics Underlies Suppressive Drug Interactions. Cell. 2009;139: 707–718. 10.1016/j.cell.2009.10.025 19914165 PMC2838386

[23] Brochado AR, Telzerow A, Bobonis J, Banzhaf M, Mateus A, Selkrig J, et al Species-specific activity of antibacterial drug combinations. Nature. 2018;559: 259–263. 10.1038/s41586-018-0278-9 29973719 PMC6219701

[24] Freschi L, Vincent AT, Jeukens J, Emond-Rheault J-G, Kukavica-Ibrulj I, Dupont M-J, et al The Pseudomonas aeruginosa Pan-Genome Provides New Insights on Its Population Structure, Horizontal Gene Transfer, and Pathogenicity. Genome Biol Evol. 2019;11: 109–120. 10.1093/gbe/evy259 30496396 PMC6328365

[25] Pena-Miller R, Laehnemann D, Jansen G, Fuentes-Hernandez A, Rosenstiel P, Schulenburg H, et al When the Most Potent Combination of Antibiotics Selects for the Greatest Bacterial Load: The Smile-Frown Transition. PLoS Biol. 2013;11: e1001540 10.1371/journal.pbio.1001540 23630452 PMC3635860

[26] Chevereau G, Bollenbach T. Systematic discovery of drug interaction mechanisms. Molecular Systems Biology. 2015;11: 807 10.15252/msb.20156098 25924924 PMC4422561

[27] White RL, Burgess DS, Manduru M, Bosso JA. Comparison of three different in vitro methods of detecting synergy: time-kill, checkerboard, and E test. Antimicrob Agents Chemother. 1996;40: 1914–1918. 10.1128/AAC.40.8.1914 8843303 PMC163439

[28] Sopirala MM, Mangino JE, Gebreyes WA, Biller B, Bannerman T, Balada-Llasat J-M, et al Synergy Testing by Etest, Microdilution Checkerboard, and Time-Kill Methods for Pan-Drug-Resistant Acinetobacter baumannii. Antimicrobial Agents and Chemotherapy. 2010;54: 4678–4683. 10.1128/AAC.00497-10 20713678 PMC2976112

[29] Hocquet D, Dehecq B, Bertrand X, Plésiat P. Strain-Tailored Double-Disk Synergy Test Detects Extended-Spectrum Oxacillinases in Pseudomonas aeruginosa. J Clin Microbiol. 2011;49: 2262–2265. 10.1128/JCM.02585-10 21450950 PMC3122726

[30] Kiraz N, Dag I, Yamac M, Kiremitci A, Kasifoglu N, Oz Y. Synergistic Activities of Three Triazoles with Caspofungin against Candida glabrata Isolates Determined by Time-Kill, Etest, and Disk Diffusion Methods. Antimicrobial Agents and Chemotherapy. 2010;54: 2244–2247. 10.1128/AAC.01527-09 20194697 PMC2863687

[31] Lamer J, Vincelette J. Quantitative study of the interaction between two antibiotics by agar diffusion. Journal of Antimicrobial Chemotherapy. 1988;21: 345–354. 10.1093/jac/21.3.345 3360692

[32] Greco WR, Bravo G, Parsons JC. The search for synergy: a critical review from a response surface perspective. Pharmacol Rev. 1995;47: 331 7568331

[33] Naghmouchi K, Le Lay C, Baah J, Drider D. Antibiotic and antimicrobial peptide combinations: synergistic inhibition of Pseudomonas fluorescens and antibiotic-resistant variants. Research in Microbiology. 2012;163: 101–108. 10.1016/j.resmic.2011.11.002 22172555

[34] Odds FC. Synergy, antagonism, and what the chequerboard puts between them. Journal of Antimicrobial Chemotherapy. 2003;52: 1–1. 10.1093/jac/dkg301 12805255

[35] Avery LM, Nicolau DP. Feasibility of routine synergy testing using antibiotic gradient diffusion strips in the clinical laboratory. Journal of Antimicrobial Chemotherapy. 2018;73: 2264–2265. 10.1093/jac/dky165 29746659

[36] Hooton TM. Uncomplicated Urinary Tract Infection. New England Journal of Medicine. 2012;366: 1028–1037. 10.1056/NEJMcp1104429 22417256

[37] Public Health Agency of Sweden, National Veterinary Institute of Sweden. Swedres-Svarm 2017. Consumption of antibiotics and occurrence of resistance in Sweden. Solna/Uppsala: Folkhälsomyndigheten; 2017 p. 118.

[38] Meletiadis J, Pournaras S, Roilides E, Walsh TJ. Defining Fractional Inhibitory Concentration Index Cutoffs for Additive Interactions Based on Self-Drug Additive Combinations, Monte Carlo Simulation Analysis, and In Vitro-In Vivo Correlation Data for Antifungal Drug Combinations against Aspergillus fumigatus. Antimicrobial Agents and Chemotherapy. 2010;54: 602–609. 10.1128/AAC.00999-09 19995928 PMC2812160

[39] Deshpande D, Srivastava S, Nuermberger E, Pasipanodya JG, Swaminathan S, Gumbo T. Concentration-Dependent Synergy and Antagonism of Linezolid and Moxifloxacin in the Treatment of Childhood Tuberculosis: The Dynamic Duo. Clin Infect Dis. 2016;63: S88–S94. 10.1093/cid/ciw473 27742639 PMC5064154

[40] Rezzoagli C, Archetti M, Mignot I, Baumgartner M, Kümmerli R. Combining antibiotics with antivirulence compounds can have synergistic effects and reverse selection for antibiotic resistance in Pseudomonas aeruginosa. PLOS Biol. 2020;18: e3000805 10.1371/journal.pbio.3000805 32810152 PMC7433856

[41] Spratt BG. Distinct penicillin binding proteins involved in the division, elongation, and shape of Escherichia coli K12. Proc Natl Acad Sci U S A. 1975;72: 2999–3003. 10.1073/pnas.72.8.2999 1103132 PMC432906

[42] Rylander M, Brorson JE, Johnsson J, Norrby R. Comparison between agar and broth minimum inhibitory concentrations of cefamandole, Cefoxitin, and cefuroxime. Antimicrob Agents Chemother. 1979;15: 572–579. 10.1128/aac.15.4.572 464588 PMC352713

[43] Yeh P, Tschumi AI, Kishony R. Functional classification of drugs by properties of their pairwise interactions. Nat Genet. 2006;38: 489–494. 10.1038/ng1755 16550172

[44] Rodriguez de Evgrafov M, Gumpert H, Munck C, Thomsen TT, Sommer MOA. Collateral Resistance and Sensitivity Modulate Evolution of High-Level Resistance to Drug Combination Treatment in Staphylococcus aureus. Mol Biol Evol. 2015;32: 1175–1185. 10.1093/molbev/msv006 25618457

[45] Chevereau G, Dravecká M, Batur T, Guvenek A, Ayhan DH, Toprak E, et al Quantifying the Determinants of Evolutionary Dynamics Leading to Drug Resistance. PLoS Biol. 2015;13: e1002299 10.1371/journal.pbio.1002299 26581035 PMC4651364

[46] Miller C, Thomsen LE, Gaggero C, Mosseri R, Ingmer H, Cohen SN. SOS Response Induction by ß-Lactams and Bacterial Defense Against Antibiotic Lethality. Science. 2004;305: 1629–1631. 10.1126/science.1101630 15308764

[47] Gutierrez A, Laureti L, Crussard S, Abida H, Rodríguez-Rojas A, Blázquez J, et al β-Lactam antibiotics promote bacterial mutagenesis via an RpoS-mediated reduction in replication fidelity. Nat Commun. 2013;4: 1610 10.1038/ncomms2607 23511474 PMC3615471

[48] Mitosch K, Rieckh G, Bollenbach T. Temporal order and precision of complex stress responses in individual bacteria. Molecular Systems Biology. 2019;15: e8470 10.15252/msb.20188470 30765425 PMC6375286

[49] Montesano R, Mouron P, Amherdt M, Orci L. Collagen matrix promotes reorganization of pancreatic endocrine cell monolayers into islet-like organoids. Journal of Cell Biology. 1983;97: 935–939. 10.1083/jcb.97.3.935 6350323 PMC2112577

[50] Gjorevski N, Sachs N, Manfrin A, Giger S, Bragina ME, Ordóñez-Morán P, et al Designer matrices for intestinal stem cell and organoid culture. Nature. 2016;539: 560–564. 10.1038/nature20168 27851739

[51] Wlodkowic D, Cooper JM. Tumors on chips: oncology meets microfluidics. Current Opinion in Chemical Biology. 2010;14: 556–567. 10.1016/j.cbpa.2010.08.016 20832352

[52] Fatsis-Kavalopoulos N, O’Callaghan P, Xie B, Hernández Vera R, Idevall-Hagren O, Kreuger J. Formation of precisely composed cancer cell clusters using a cell assembly generator (CAGE) for studying paracrine signaling at single-cell resolution. Lab Chip. 2019;19: 1071–1081. 10.1039/c8lc01153b 30783638

[53] Wistrand-Yuen P, Malmberg C, Fatsis-Kavalopoulos N, Lübke M, Tängdén T, Kreuger J. A Multiplex Fluidic Chip for Rapid Phenotypic Antibiotic Susceptibility Testing. Bomberger JM, editor. mBio. 2020;11: e03109–19, /mbio/11/1/mBio.03109-19.atom. 10.1128/mBio.03109-19 32098819 PMC7042698

[54] Baltekin Ö, Boucharin A, Tano E, Andersson DI, Elf J. Antibiotic susceptibility testing in less than 30 min using direct single-cell imaging. Proc Natl Acad Sci USA. 2017;114: 9170 10.1073/pnas.1708558114 28790187 PMC5576829

[55] Russ D, Kishony R. Additivity of inhibitory effects in multidrug combinations. Nat Microbiol. 2018;3: 1339–1345. 10.1038/s41564-018-0252-1 30323252 PMC6295580

[56] Bliss CI. The Toxicity of Poisons Applied Jointly1. Annals of Applied Biology. 1939;26: 585–615. 10.1111/j.1744-7348.1939.tb06990.x

[57] Miles AA, Misra SS, Irwin JO. The estimation of the bactericidal power of the blood. J Hyg (Lond). 1938;38: 732–749. 20475467 10.1017/s002217240001158x PMC2199673

