# Supplementary figures and images for: CombiANT: Antibiotic interaction testing made easy
Source: PLoS Biol. 2020 Sep 17;18(9):e3000856. doi: 10.1371/journal.pbio.3000856 (PMC7524002; doi:10.1371/journal.pbio.3000856)

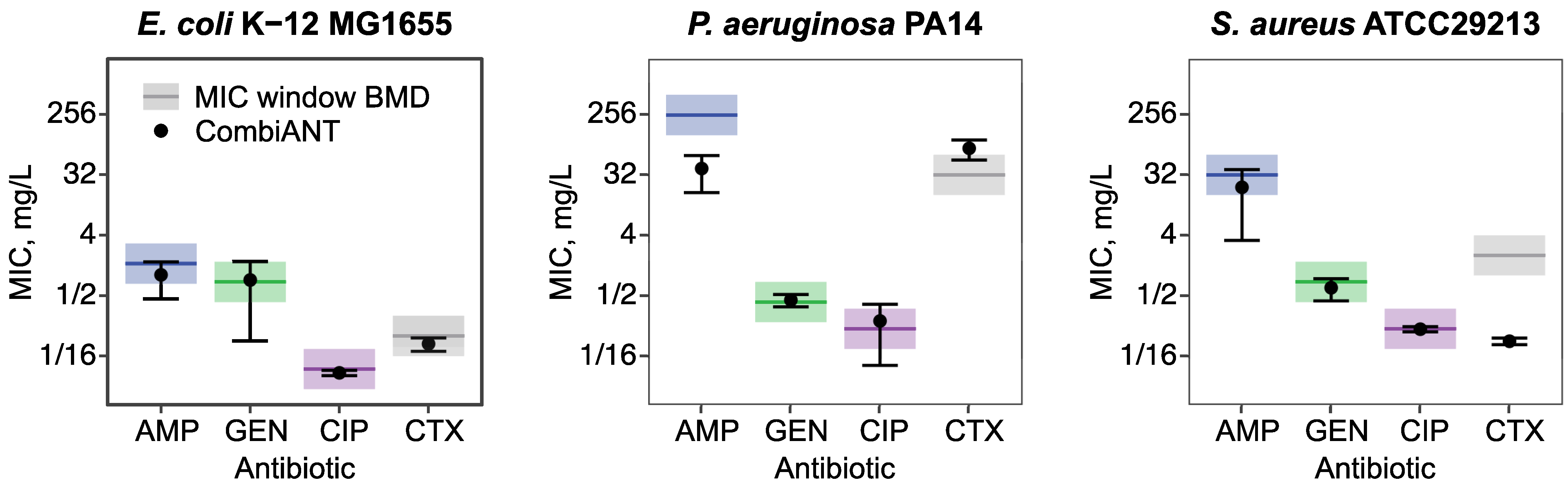

Supplement: S1 Fig — Comparison of the MIC values obtained with CombiANT as part of the synergy calculations with those obtained from BMD. Data of reference strains E. coli, P. aeruginosa, and S. aureus tested against the antibiotics AMP, CIP, GEN, and CTX. CombiANT MIC predictions (indicated by solid points, mean ± SD, n = 18, 3 biological replicates of 6 technical replicates each) fall within the log2 dilution window of the BMD assay (indicated by the shaded regions), supporting the physics diffusion model. An accurate determination of MIC for P. aeruginosa by BMD was not possible for AMP and CTX due to extensive cell elongation preceding cell death. Numerical values for this figure are available in S1 Data. BMD, broth microdilution. (TIF) [file pbio.3000856.s001.tif]

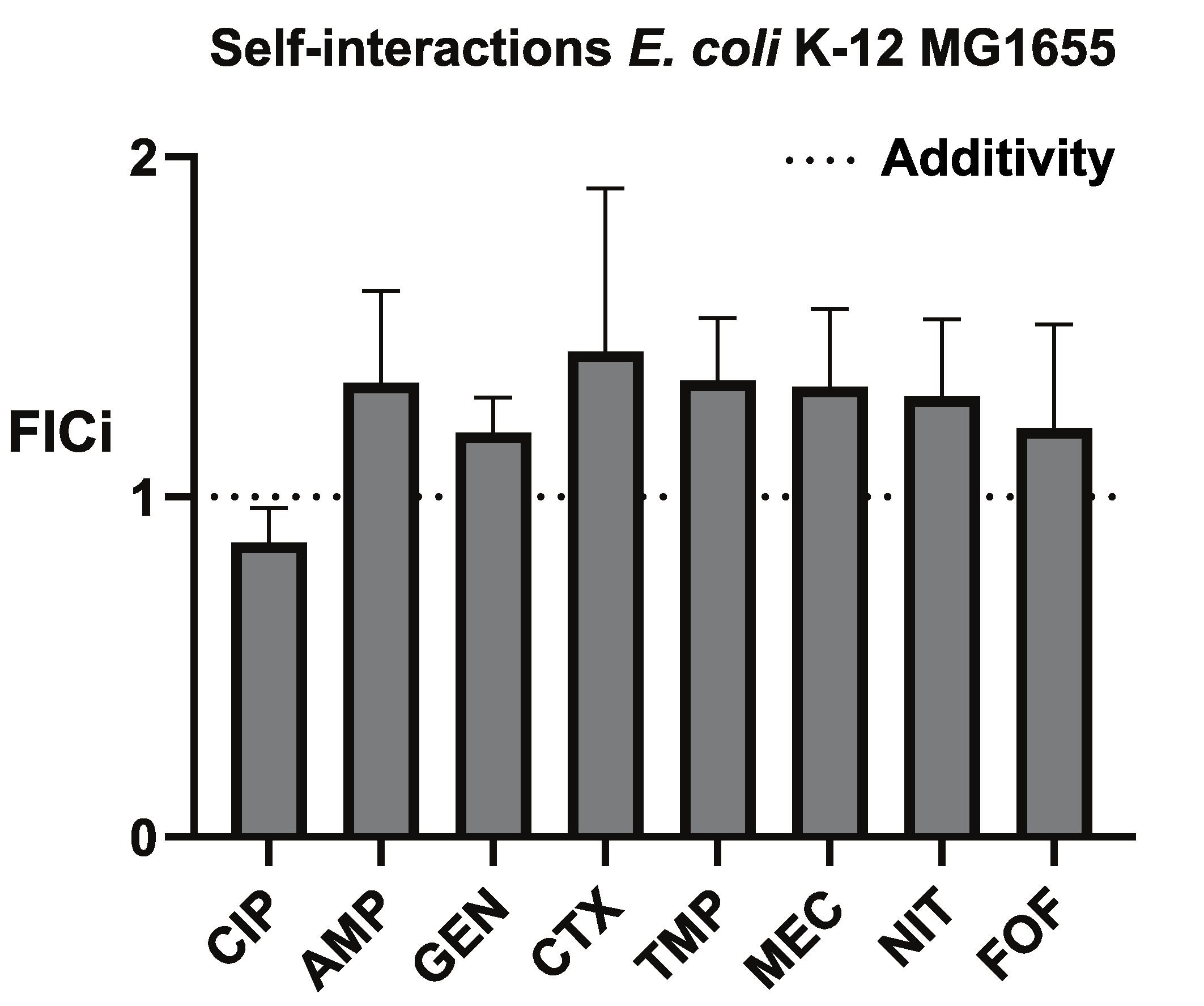

Supplement: S2 Fig — FICi for all self-interactions for the 8 antibiotics AMP, CIP, CTX, GEN, FOF, NIT, MEC, and TMP. Bars indicate average values and SD from 3 biological replicates. The dotted line indicates additivity, i.e., FICi = 1. All interactions were additive in nature and showed no statistically significant difference from FICi = 1 according to one-sample Wilcoxon signed rank test. Numerical values for this figure are available in S1 Data. FICi, fractional inhibitory concentration index (TIF) [file pbio.3000856.s002.tif]

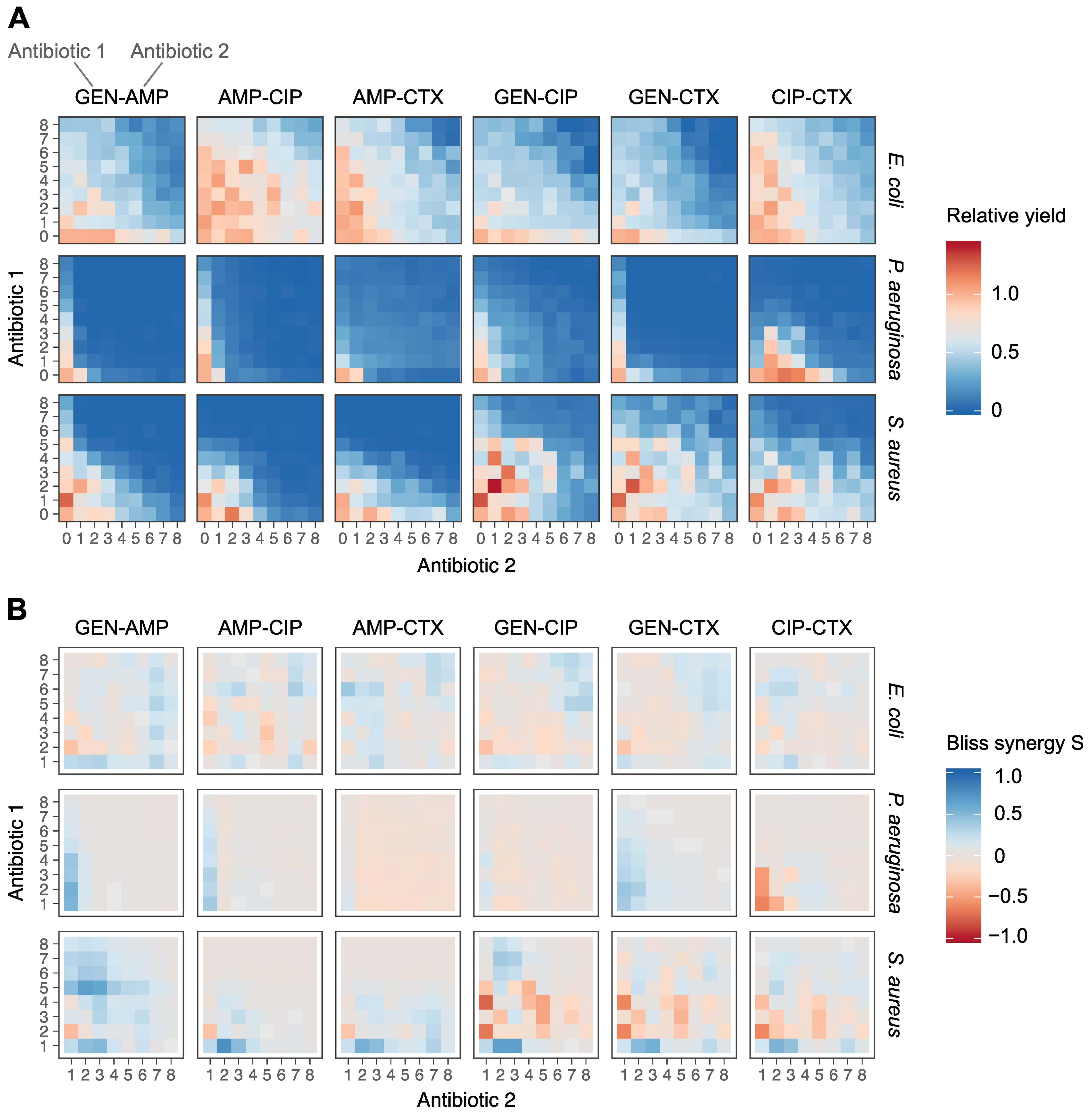

Supplement: S3 Fig — (A) Antibiotic checkerboard data with linear concentrations ranges for E. coli, P. aeruginosa, and S. aureus tested against the antibiotics AMP, CIP, GEN, and CTX. Each antibiotic was tested with 8 equally spaced linearly increasing concentrations (denoted 1–8) between zero and the MIC for the respective species. Data are average growth values of 3 biological replicates relative to untreated controls. (B) Dose-dependent synergy profiles according to a Bliss independence additive model. Numerical values for this figure are available in S1 Data. (TIF) [file pbio.3000856.s003.tif]

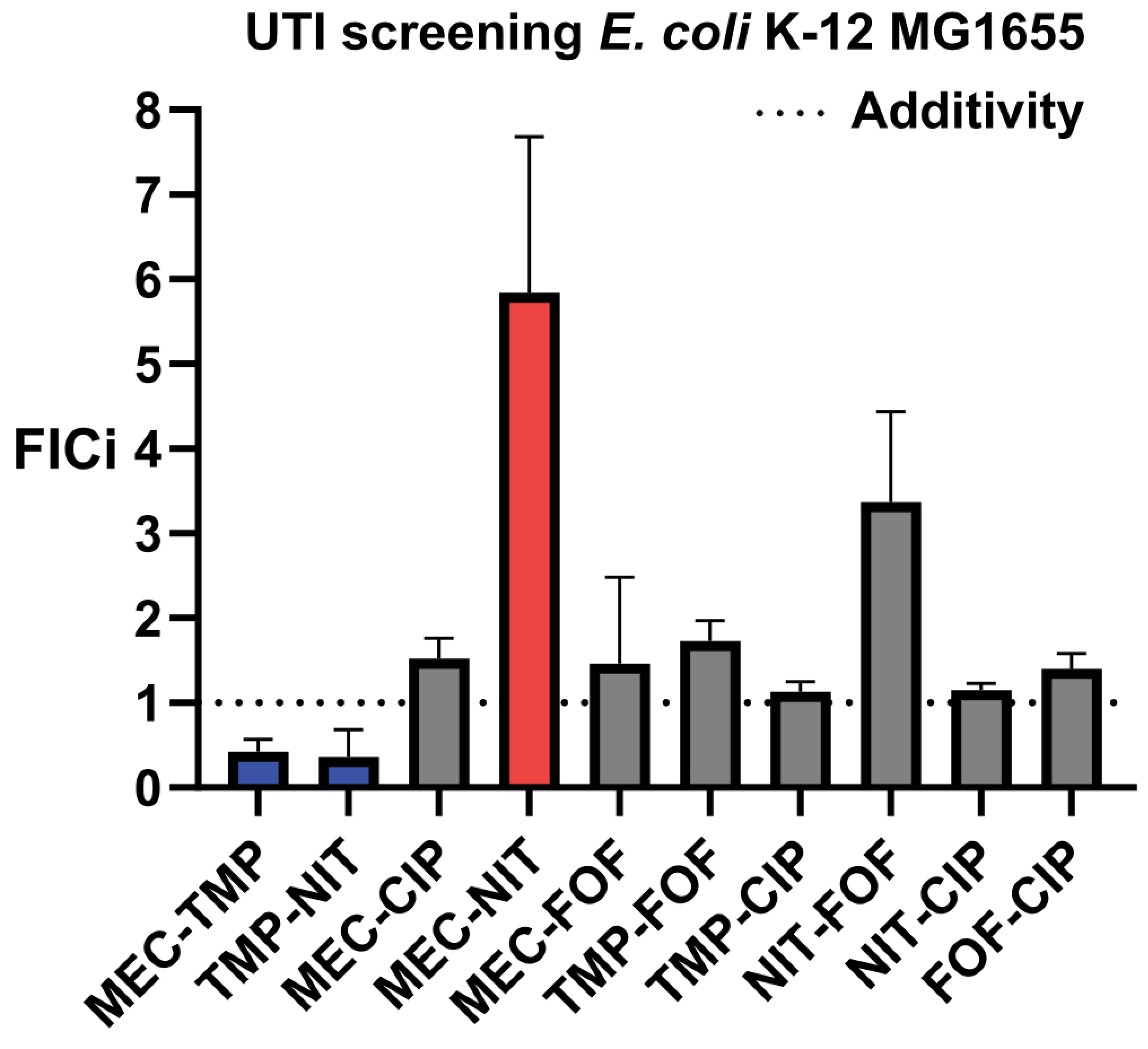

Supplement: S4 Fig — FICi for all pairwise combinations of the 5 antibiotics CIP, FOF, NIT, MEC, and TMP. Bars indicate average values and SD from 3 biological replicates. The dotted line indicates additivity, i.e., FICi = 1. Synergy with FICi < 0.5 is indicated in blue, antagonism with FICi > 4 is indicated in red. One-sample Wilcoxon signed rank test against FICi = 1, *P < 0.05. Numerical values for this figure are available in S1 Data. FICi, fractional inhibitory concentration index (TIF) [file pbio.3000856.s004.tif]

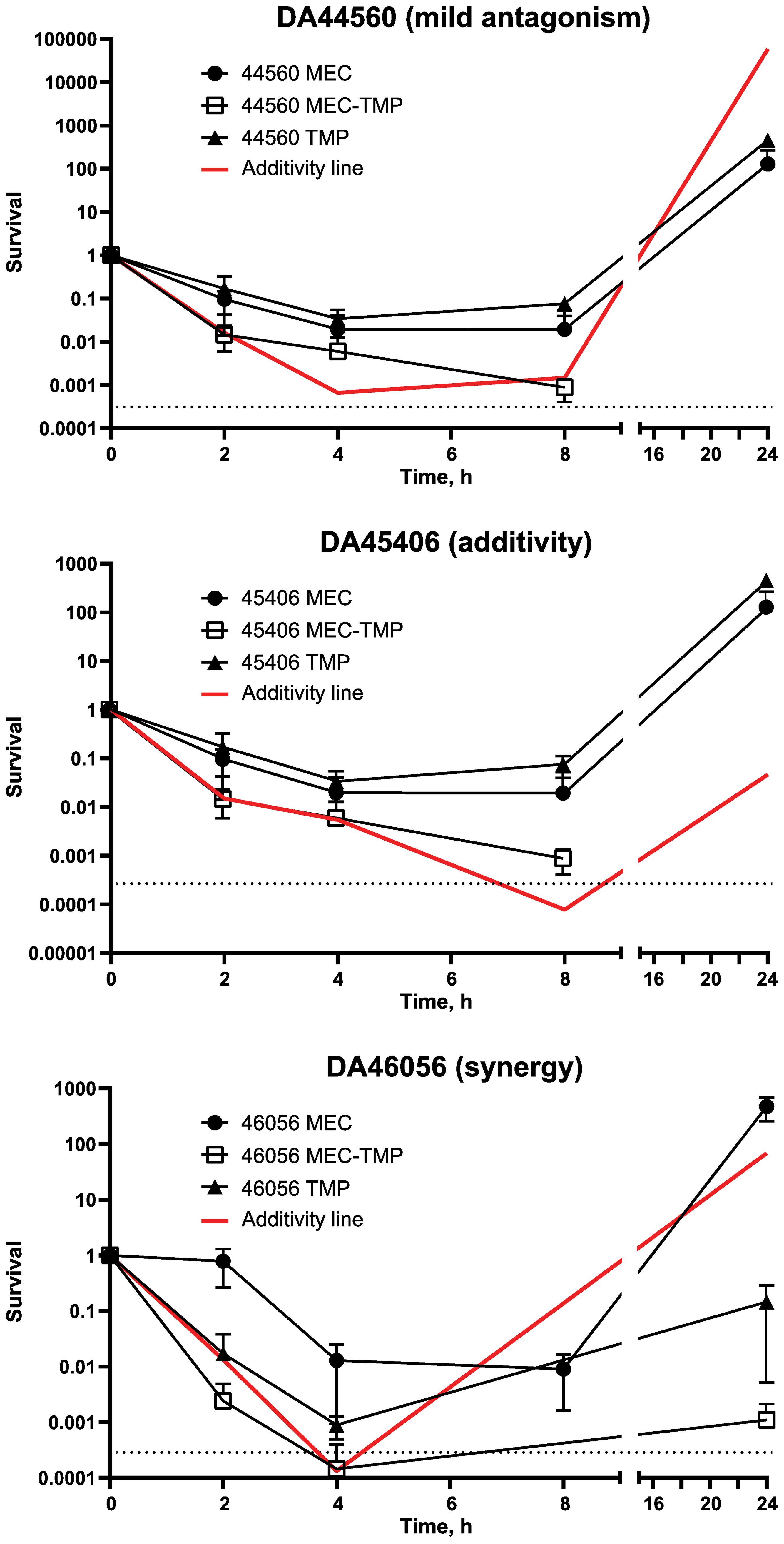

Supplement: S5 Fig — Time-kill assay on the UTI strains DA44560, DA45406, and DA46056. The 3 strains showed contrasting interaction profiles for the MEC-TMP combination in the CombiANT assay, which was additive for DA45406, synergistic for DA46056, and mildly antagonistic for DA44560 (although FICi < 4). Survival fraction was expressed as CFU/mL relative to time point 0 hours (mean ± SD, n = 3 biological replicates). Antibiotics were applied at isolate-adjusted MIC dosage. The circle symbols (●) denote the effect of MEC on strain survival, triangles (π) denote the effect of TMP, and open squares (ο) denote the combined effect of the MEC-TMP combination. The red lines represent the Bliss additive interaction model (calculated as the multiplication of survival rates, of the antibiotics when acting individually). Increased survival in the combination compared to the additivity line indicates antagonism, whereas the opposite indicates synergy. The dotted line shows the assay’s limit of certainty. Missing data points indicate a colony count of zero (not defined for logarithmic scales). Numerical values for this figure are available in S1 Data. CFU, colony forming units (TIF) [file pbio.3000856.s005.tif]

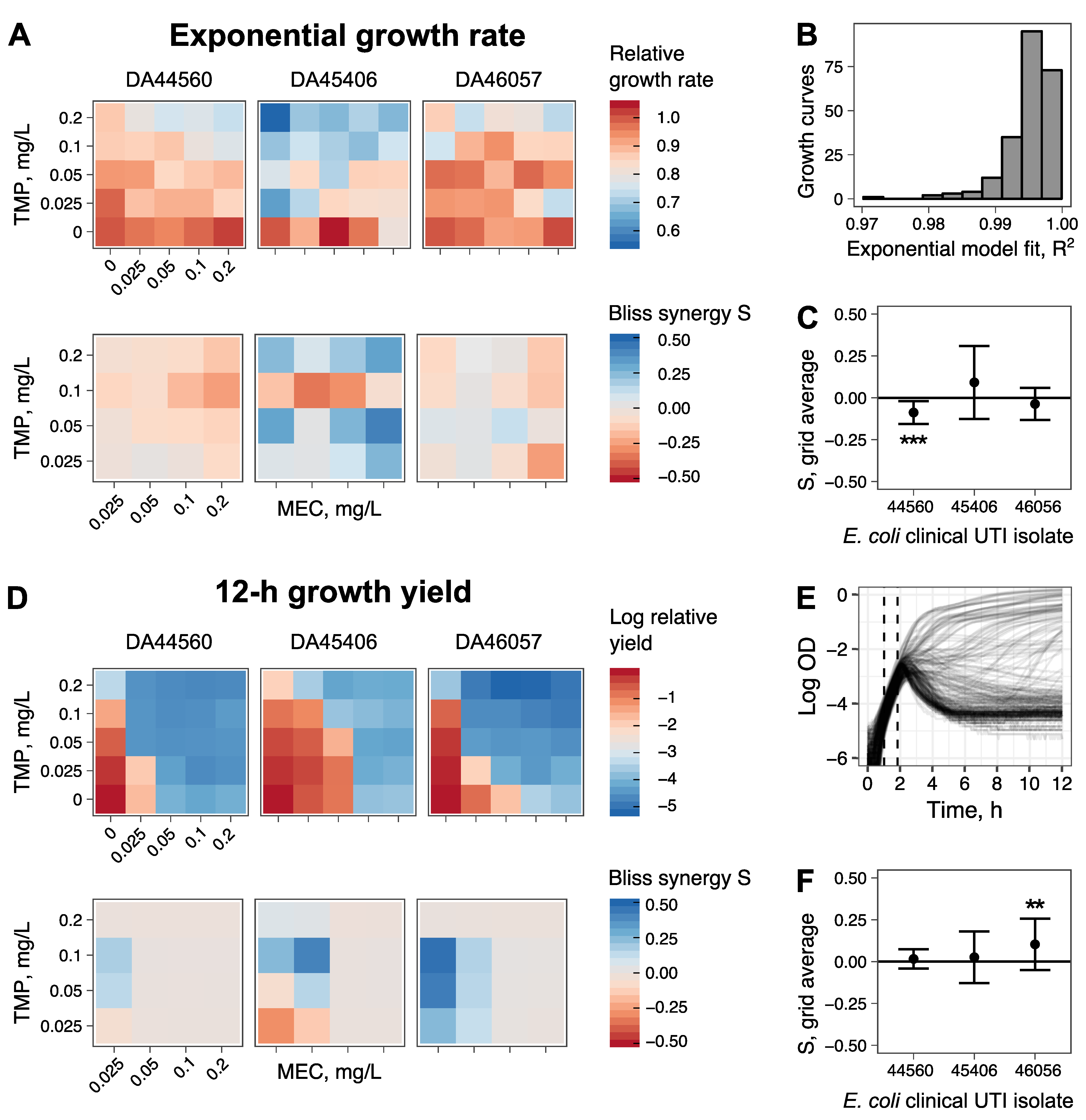

Supplement: S6 Fig — The UTI isolates DA44560, DA45406, and DA46056 were treated with low concentrations of MEC, TMP, or combinations of MEC-TMP, using a 5 × 5 checkerboard setup. The 3 strains showed contrasting interaction profiles for the MEC-TMP combination in the CombiANT assay, which was additive for DA45406, synergistic for DA46056, and mildly antagonistic for DA44560 (although FICi < 4). Growth was monitored in 4-minute intervals using OD. (A) Exponential growth rates of the checkerboard, and degree of synergy S in drug–drug combinations according to a Bliss additive model (growth rate inhibition values are added). Positive values of S indicate synergy, and negative values indicate antagonism. Values are averages of 3 biological replicates. (B) Distribution of coefficient of determination R2 for fitted exponential growth models in the measurement time window of 60 to 110 minutes after start of treatment. (C) Average degree of growth rate synergy across the concentration grid (mean ± SD, n = 16 combinations). (D) Growth yields after 12 hours of treatment, and calculated degree of synergy according to a Bliss additive model (relative yields are multiplied). Values are averages of 3 biological replicates. (E) Logarithmic presentation of underlying growth curves. The dotted lines show the measurement window for exponential growth rates in panel A. (F) Average degree of growth yield synergy across the concentration grid (mean ± SD, n = 16 combinations). MEC-TMP additivity in DA45406 is replicated by growth rate and growth yield measurements. MEC-TMP synergy in DA46056 is apparent in growth yields, but not growth rates. Mild antagonism of MEC-TMP in DA44560 is replicated in growth rates, but not growth yields. Numerical values for this figure are available in S1 Data. OD, optical density at 600 nm (TIF) [file pbio.3000856.s006.tif]

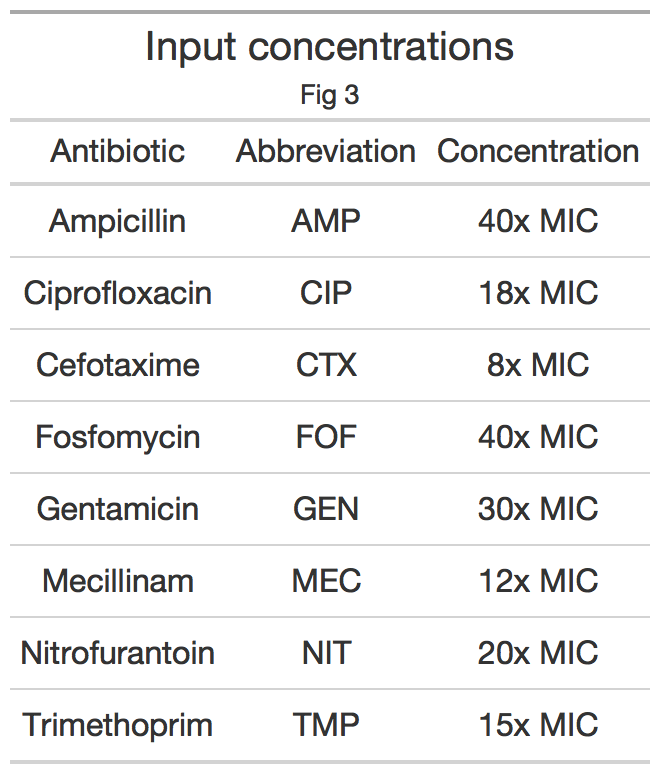

Supplement: S1 Table — MIC-based initial concentrations for the technical calibration with reference strains E. coli, P. aeruginosa, and S. aureus and the antibiotics AMP, CIP, GEN, and CTX. (PNG) [file pbio.3000856.s009.png]

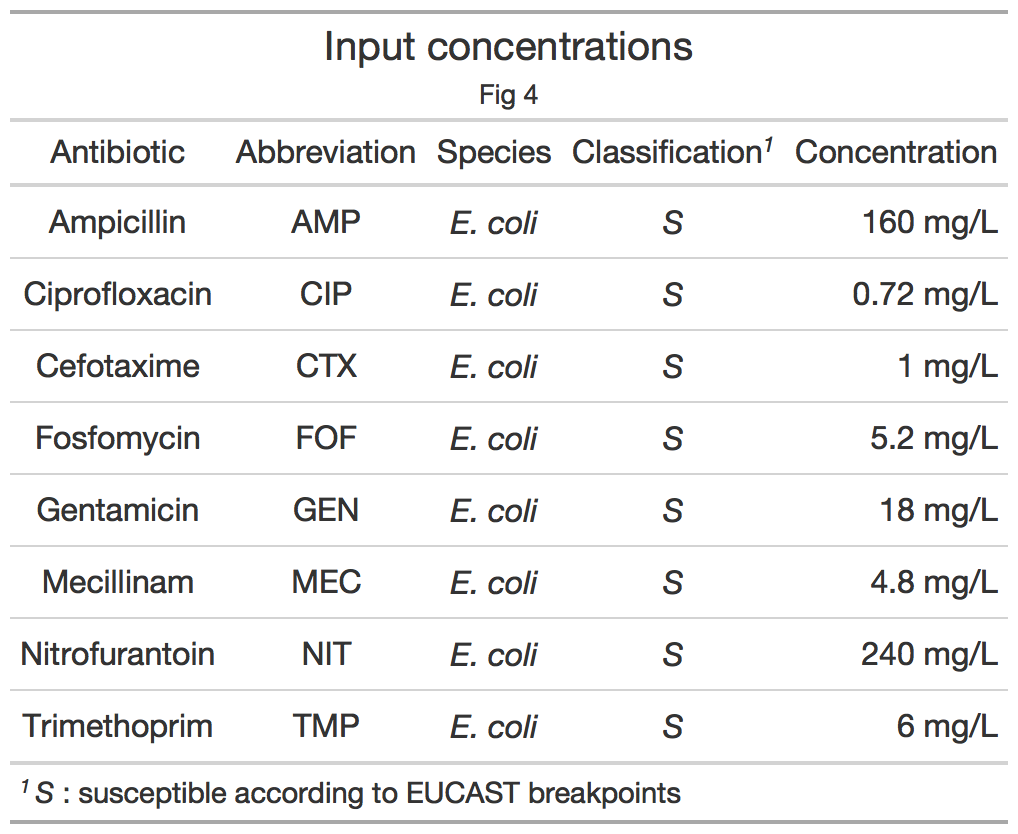

Supplement: S2 Table — Breakpoint-based initial concentrations, as applied for the synergy screening of the E. coli UTI isolates. (PNG) [file pbio.3000856.s010.png]

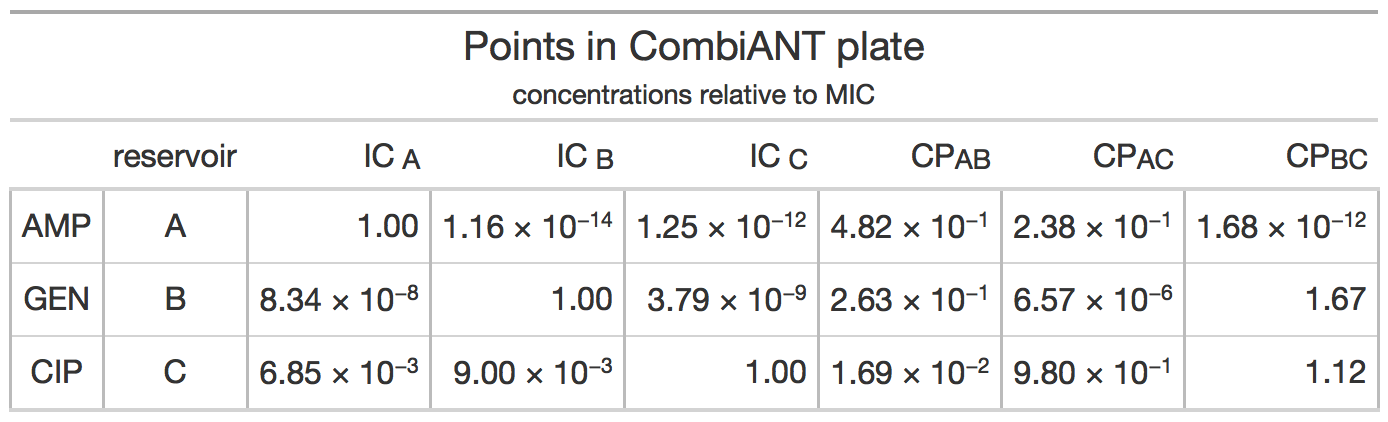

Supplement: S3 Table — Concentrations of AMP, CIP, and GEN in a CombiANT plate of E. coli K-12 MG1665. Concentrations in every point are expressed as fold of MIC for that specific antibiotic in a broth microdilution. (PNG) [file pbio.3000856.s011.png]
