# Supplementary material for: CombiANT: Antibiotic interaction testing made easy
Source: PLoS Biol. 2020 Sep 17;18(9):e3000856. doi: 10.1371/journal.pbio.3000856 (PMC7524002; doi:10.1371/journal.pbio.3000856)
Supplement: S1 Code — (DOCX) [file pbio.3000856.s008.docx]

function [plates,elapsed_time] = combiANT_1(analysis,antibiotics,antibiotics_MW)

%UNTITLED2 Summary of this function goes here

% Detailed explanation goes here

tic;

counter=0;

plates=struct;

%convert the image analysis data points to physics model data points

conversion_factor=41/1000;

size(analysis,1);

for n=1:size(analysis,1)

%extract coordinate data from analysis

coordinatesrowcell= cell2mat(analysis(:,12:23));

coordinatesrow=(coordinatesrowcell(n,:));

coordinates=[coordinatesrow(1),coordinatesrow(2);coordinatesrow(3),coordinatesrow(4);coordinatesrow(5),coordinatesrow(6);coordinatesrow(7),coordinatesrow(8);coordinatesrow(9),coordinatesrow(10);coordinatesrow(11),coordinatesrow(12)];

points = coordinate_system_change(coordinates,conversion_factor,cell2mat(analysis(n,8:9)),cell2mat(analysis(n,10:11)));

plates(n).points=points;%stores all relevant coordinates in a 2x6 matrix under struct fields of plate numbers i.e. for plate 1 platepoints(1).points

end

%this has created a matrix MICFICpoints with 6 rows,

%MICA, MICB, MICC, FICAB, FICAC, FICBC. every 2 columns is a new point because

%every pair of columns is a x,y coordinate

for k=1:size(analysis,1)%%%%%%%%%%%%%%%%%%%%%%%%%%%%%%%%%%%%%%%%%%%%%%%%%%%%%%%%%%%%%%%%%%%%%%%%%%%%%%%%%%%%%%%%%%%%%%%%%%%%%%%%%%%%%%%%%%%%%%%%%%%%%%%%%%%%%%%%%%%%%%%%%%%%%%%%%%%%%%%%

%requires a struct object antibiotics with all antibiotic coefficients in the form

%of antibiotics.AMP=1.234235

plates(k).strain=analysis(k,1);

plates(k).ABa=analysis(k,2);%putting the data in a struct for later

plates(k).ABb=analysis(k,3);

plates(k).ABc=analysis(k,4);

% following line reads initial C and what antibiotic from dataset and

% creates the landscape for that plate

map = combiant_create_landscape( [antibiotics.(string(analysis(k,2))),antibiotics.(string(analysis(k,3))),antibiotics.(string(analysis(k,4)))],[cell2mat(analysis(k,5)),cell2mat(analysis(k,6)),cell2mat(analysis(k,7))] );

plates(k).concentrations = combiant_get_concentrations(plates(k).points,map); %gets the concentrations from the landscape using the points for this iterations plate and store it in the struct

%requires a molecular weight struct to give FIC and MIC in mg/L

[FIC ] = combiant_report_MIC_FIC_2(plates(k).concentrations,[antibiotics_MW.(string(analysis(k,2))),antibiotics_MW.(string(analysis(k,3))),antibiotics_MW.(string(analysis(k,4)))]); %calculate MICS and FICS

plates(k).FICAB=FIC(1,2);

plates(k).FICAC=FIC(1,3);

plates(k).FICBC=FIC(2,3);

function [ mic,fic ] = combiant_analyze( diffusion_coef,initial_concentrations,reading_points)

%full analysis of combiant data

%just give diffusion coefficient, initial concentrations and points along

%the inhibition zone

%always do A B C and obtain reading with A horizontal and on the bottom

%input coordinates in 2 column format

map = combiant_create_landscape( diffusion_coef,initial_concentrations );

concentrations = combiant_get_concentrations(reading_points,map);

[ mic, fic ] = combiant_report_MIC_FIC(concentrations);

end

function [ map ] = combiant_create_landscape( coeffs,cons )

%you give this the coefficients of the antibiotics you used A B and then C (calibrated for)

%and the initial concentrations for them. From that it runs the simulation and

%calculates the antibiotic landscape.

%%%%%Attention: in images the A pocket should always be on the bottom and horizontal

%%%%%Attention: concentrations need to be in mol/m3

import com.comsol.model.util.*

model=mphload('combiant1.mph');

%for antibiotic A

model.param.set('initconc1',cons(1));

model.param.set('initconc2',0);

model.param.set('initconc3',0);

model.param.set('difcoef',coeffs(1));

model.sol('sol1').runAll; % solve

c1=mpheval(model,'c','t',[24]); %get the concentration of A for all nodes at t=24h

cA=c1.d1; %store the concentrations of A for all nodes

coord=c1.p; %store the coordinates of all nodes

%for antibiotic B

model.param.set('initconc2',cons(2));

model.param.set('initconc1',0);

model.param.set('initconc3',0);

model.param.set('difcoef',coeffs(2));

model.sol('sol1').runAll; % solve

c2=mpheval(model,'c','t',[24]);

cB=c2.d1;

%for antibiotic C

model.param.set('initconc3',cons(3));

model.param.set('difcoef',coeffs(3));

model.param.set('initconc1',0);

model.param.set('initconc2',0);

model.sol('sol1').runAll; % solve

c3=mpheval(model,'c','t',[24]);

cC=c3.d1;

concentrations=cat(1,cA,cB,cC); %matrix with the concentrations of all 3 ABs for all nodes

map=struct('coordinates',coord,'concentration_A_B_C',concentrations);

end

function [ map ] = combiant_data_extraction( coeffs,cons )

%you give this the coefficients of the antibiotics you used A B and then C (calibrated for)

%and the initial concentrations for them. From that it runs the simulation and

%calculates the antibiotic landscape.

%%%%%Attention: in images, the A pocket should always be on the bottom and horizontal

import com.comsol.model.util.*

model=mphload('combiant1.mph');

%for antibiotic A

model.param.set('initconc1',cons(1));

model.param.set('difcoef',coeffs(1));

model.sol('sol1').runAll; % solve

c1=mpheval(model,'c','t',[24]); %get the concentration of A for all nodes at t=24h

cA=c1.d1; %store the concentrations of A for all nodes

coord=c1.p; %store the coordinates of all nodes

%for antibiotic B

model.param.set('initconc2',cons(2));

model.param.set('difcoef',coeffs(2));

model.sol('sol1').runAll; % solve

c2=mpheval(model,'c','t',[24]);

cB=c2.d1;

%for antibiotic C

model.param.set('initconc3',cons(3));

model.param.set('difcoef',coeffs(3));

model.sol('sol1').runAll; % solve

c3=mpheval(model,'c','t',[24]);

cC=c3.d1;

concentrations=cat(1,cA,cB,cC); %matrix with the concentrations of all 3 ABs for all nodes

map=struct('coordinates',coord,'concentration_A_B_C',concentrations);

end

function [ conc] = combiant_get_concentrations( coordinates,map)

%map needs to be a struct generated from combiant_create_landscape

%coordinates are the points in the comsol reference system that you want to

%analyze

%calculates concentration values for multiple points

% coordinates should be the following point with that order

% MIC.A

% MIC:B

% MIC:C

% FIC:AB

% FIC:AC

% FIC:BC

for n=1:length(coordinates)

conc(:,n)=concentration_value(coordinates(n,1),coordinates(n,2),map);

end

function [ values ] = concentration_value( x,y,map )

%UNTITLED2 you give the x and y converted into the comsol coordinate system

%together with the map generated from combiant_create_landscape. The function calculates

%the concentrations of all ABs that point has from your comsol model

%

match_matrix=sqrt((map.coordinates(1,:)-x).^2 + (map.coordinates(2,:)-y).^2); %calculate point distance from every node

index=find(match_matrix==min(match_matrix)); %find the closest node

values=map.concentration_A_B_C(:,index); %get the concentration values for that node

end

end

function [ MIC, FIC ] = combiant_report_MIC_FIC(concentrations)

%reporting of values use the concentrations reported from

%combiant_get_concentrations

%MIC table is a vector with 3 values for the 3 MIC (A B C)

%FIC table is as follows

% A B C

% A 0 FICAB FICAC

% B FICAB 0 FICBC

% C FICAC FICBC 0

MIC(1)=concentrations(1,1);

MIC(2)=concentrations(2,2);

MIC(3)=concentrations(3,3);

FIC=zeros(3,3); %initialize FIC

FIC(1,2)=MIC(1)/concentrations(1,4)+MIC(2)/concentrations(2,4);

FIC(2,1)=MIC(1)/concentrations(1,4)+MIC(2)/concentrations(2,4);

FIC(1,3)=MIC(1)/concentrations(1,5)+MIC(3)/concentrations(3,5);

FIC(3,1)=MIC(1)/concentrations(1,5)+MIC(3)/concentrations(3,5);

FIC(2,3)= MIC(2)/concentrations(2,6)+MIC(3)/concentrations(3,6);

FIC(3,2)= MIC(2)/concentrations(2,6)+MIC(3)/concentrations(3,6);

end

function [ MIC, FIC ] = combiant_report_MIC_FIC_2(concentrations,MW)

%reporting of values use the concentrations reported from

%combiant_get_concentrations

%MIC table is a vector with 3 values for the 3 MIC (A B C)

%FIC table is as follows

% concentration from the physics model comes in mol/m3 that is MW*c mg/L

MIC(1)=concentrations(1,1)*MW(1);

MIC(2)=concentrations(2,2)*MW(2);

MIC(3)=concentrations(3,3)*MW(3);

FIC=zeros(3,3); %initialize FIC

FIC(1,2)=(MIC(1)/(concentrations(1,4)*MW(1)))^-1 +(MIC(2)/(concentrations(2,4)*MW(2)))^-1;

FIC(1,3)=(MIC(1)/(concentrations(1,5)*MW(1)))^-1 +(MIC(3)/(concentrations(3,5)*MW(3)))^-1;

FIC(2,3)=(MIC(2)/(concentrations(2,6)*MW(2)))^-1 +(MIC(3)/(concentrations(3,6)*MW(3)))^-1;

end

function [xy_comsol] = coordinate_system_change(xy_imagej,conversion_factor,reference_point_AB,reference_point_AC)

%this function is meant to transform points in image j images

%using the plate reader into coordinates used by the comsol file combiant1

%both xy are matrices with 2 columns one for x and one for y

%the conversion factor is the mm/pixel coefficient of the photo for our camera

%it is 34mm/790pixels

%reference point is the corner between the A and C chamber that we use to

%align the 2 coordinate systems

%%%%%Attention: this assumes that the photo is originally aligned so that the A

%chamber boundary is horizontal and on the bottom of the picture

xy_comsol=xy_imagej;

xy_comsol(:,1)=xy_comsol(:,1)-2464/2;% move origin to center of plate

xy_comsol(:,2)=xy_comsol(:,2)-2464/2;

reference_point_AC(1)=reference_point_AC(1)-2464/2;

reference_point_AC(2)=reference_point_AC(2)-2464/2;

reference_point_AB(1)=reference_point_AB(1)-2464/2;

reference_point_AB(2)=reference_point_AB(2)-2464/2;

rotation_angle=atand((reference_point_AC(2)-reference_point_AB(2))/(reference_point_AB(1)-reference_point_AC(1))); %calculated to be positive when representing clockwise turn

xy_comsoln(:,1)= xy_comsol(:,1)*cosd(-1*rotation_angle) +xy_comsol(:,2)*sind(-1*rotation_angle); %apply rotation of coordinate system

xy_comsoln(:,2)=-1*xy_comsol(:,1)*sind(-1*rotation_angle) +xy_comsol(:,2)*cosd(-1*rotation_angle);

xy_comsol=xy_comsoln;

reference_point_ACn(1)=reference_point_AC(1)*cosd(-1*rotation_angle) +reference_point_AC(2)*sind(-1*rotation_angle);

reference_point_ACn(2)=-1*reference_point_AC(1)*sind(-1*rotation_angle) +reference_point_AC(2)*cosd(-1*rotation_angle);%shift coordinates of ac corner

reference_point_AC=reference_point_ACn;

xy_comsol(:,1)=xy_comsol(:,1)+2464/2;%move origin back to top left corner

xy_comsol(:,2)=xy_comsol(:,2)+2464/2;

reference_point_AC(1)=reference_point_AC(1)+2464/2;

reference_point_AC(2)=reference_point_AC(2)+2464/2;

%change the y axis to count from bottom to top sibliroma os pros 2464

xy_comsol(:,2)=2464- xy_comsol(:,2);

reference_point_AC(2)=2464 -reference_point_AC(2);

reference_point_AC=reference_point_AC * conversion_factor; %converts the reference points to mm coordinates

xy_comsol=xy_comsol *conversion_factor; %convert image j pixel coordinates into mm coordinates (scale the picture)

x_shift= reference_point_AC(1)- 89.21 ; %calculate how much to shift in the x by subtracting the reference point coordinates from the model coordinates of that point

y_shift= reference_point_AC(2)- 197.45; %same but for y

xy_comsol(:,1)= xy_comsol(:,1) -x_shift; %apply move of coordinate system

xy_comsol(:,2)=xy_comsol(:,2) -y_shift;

end
