# Supplementary material for: CombiANT: Antibiotic interaction testing made easy
Source: PLoS Biol. 2020 Sep 17;18(9):e3000856. doi: 10.1371/journal.pbio.3000856 (PMC7524002; doi:10.1371/journal.pbio.3000856)
Supplement: S1 Text — The calibration protocol for the use of new media-antibiotic combination in the CombiANT assay. (DOCX) [file pbio.3000856.s012.docx]

**Calibration protocol**

For every antibiotic that was used in this study, a one-time calibration of the physics model is needed. All potential new antibiotics used with CombiANT assays will also require the same calibration step. We elected to not try and model diffusion coefficients theoretically. Instead we calculated all diffusion coefficients experimentally through an iterative process. Minimum inhibitory concentrations for an antibiotic were determined using broth microdilution, then different concentrations of this antibiotic were put in the reservoirs of CombiANT and incubated for 24 hours. At the end of the 24 hours, inhibition zones form around the insert reservoirs. The edge of those zones has: a known concentration (the MIC calculated with the BMD), after a known time span (24 hours), from a known starting concentration in the reservoirs. Using this information an iterative FEM model calculated the experimental diffusion coefficient that would match the above parameters. The validity of this approach was verified by comparing the MIC data the experimental coefficients produced in the MG1665 to the actual data from broth microdilution (as shown in S1 Fig).

For every antibiotic tested, a suitable reference strain is selected. In this study, all calibrations were performed with *E. coli* strain K-12 MG1665. Below are the steps of the calibration protocol:

1. MIC of the antibiotic against the reference strain is determined using a broth microdilution assay (BMD).
2. A CombiANT assay is prepared in triplicate. The three reservoirs are loaded with 10 ×, 20 ×, and 40 × antibiotic concentrations in Mueller-Hinton agar.
3. Following the protocol of the CombiANT assay, a final layer of agar is poured on the three inserts and after it solidifies, a population of the reference strain is inoculated according to the protocol.
4. After 24 h, inhibition zones are formed on the outside of the insert as shown on Fig 2.

The edge of the concentration zones on the outside of the inserts, corresponds to the MIC of the antibiotic in use against the strain. The FEM concertation model’s diffusion coefficient is tuned iteratively until the predicted concentration at the edge of all three inhibition zones matches the experimental value determined by the BMD. Once the tuning of the diffusion coefficient is complete, the model is tested against the two remaining replicates of the tuning assay. If variability in the MIC prediction is less than 10% of the experimental one for both remaining assays, then the calibration is completed. Following the diffusion model calibration, that antibiotic can be used in all experimental assays. The recommended initial antibiotic concentration is calculated as the concentration that would result in an inhibition zone of 5 mm.
