# Supplementary material for: CombiANT: Antibiotic interaction testing made easy
Source: PLoS Biol. 2020 Sep 17;18(9):e3000856. doi: 10.1371/journal.pbio.3000856 (PMC7524002; doi:10.1371/journal.pbio.3000856)
Supplement: S2 Text — The execution protocol for the CombiANT assay. (DOCX) [file pbio.3000856.s013.docx]

**Protocol for CombiANT assay**

1. Outside the use pipeline: Prepare the CombiANT inserts.
   1. Put a CombiANT insert in a sterile petri dish
   2. Consult S1 Table (MIC-based high-resolution determination) or S2 Table (breakpoint-based determination) for input antibiotic concentrations
   3. Dilute antibiotic to input concentration in liquid autoclaved Mueller-Hinton agar (temperature 50 to 65°C)
   4. Add 0.5 mL of antibiotic agar to assigned chamber of the CombiANT insert
   5. Add petri dish lid and refrigerate on a level surface to allow setting of the agar. At 4°C, the loaded inserts are stable for at least one week
2. Before use: Grow a dense overnight culture of target strain from a single colony in Mueller-Hinton broth.
3. In use: Activate CombiANT inserts by overcasting with Mueller-Hinton agar. For a standard 90 mm petri dish, add 25 mL of Mueller-Hinton agar.
4. Let the agar set for 2-3 h at room temperature. At this stage, the plates may not be moved until the agar sets.
5. Dilute the dense bacterial culture to 0.5 McFarland.
6. Using a sterile cotton swab, inoculate plate surface with bacteria to obtain lawn growth, in accordance with EUCAST guidelines for disk diffusion tests v.8.0. It is important to streak densely and in three directions.
7. Incubate plates for 24 h.
8. Take a picture of the plate and identify the CP and IC points.
9. Input the data into the analysis algorithm.
